# Taxonomic Notes on the ‘Mahat’ (*Artocarpus lacucha* and *A. thailandicus*, Moraceae) Species Complex in Thailand

**DOI:** 10.3390/plants9030391

**Published:** 2020-03-22

**Authors:** Chaiwat Aneklaphakij, Somnuk Bunsupa, Yotsawate Sirichamorn, Bhanubong Bongcheewin, Veena Satitpatipan

**Affiliations:** 1 Department of Pharmacognosy, Faculty of Pharmacy, Mahidol University, Bangkok 10400, Thailand; chaiwat.ane@mahidol.ac.th (C.A.); somnuk.bun@mahidol.ac.th (S.B.); 2 Department of Biology, Faculty of Science, Silpakorn University, Sanam Chandra Palace Campus, Nakhon Pathom 73000, Thailand; sirichamorn_y@silpakorn.edu; 3 Department of Pharmaceutical Botany, Faculty of Pharmacy, Mahidol University, Bangkok 10400, Thailand; bhanubong.boc@mahidol.ac.th

**Keywords:** *Artocarpus lacucha*, *Artocarpus lakoocha*, *Artocarpus thailandicus*, leaf morphology, monkey jack, pharmaceutical product registration, phylogeny, phytochemistry, quality control, SEM

## Abstract

‘Mahat’ is a well-known medicinal plant utilized in Thailand. The Thai name ‘Mahat’ has been used in many scientific articles for years. However, it is, unpredictably, a homonym of two scientific names in Flora of Thailand, i.e., *A. lacucha* and *A. thailandicus*. Additionally, both species are complex due to their high morphological variation. This causes difficulties in species identification especially when this Thai name is referred to as the scientific name for research publication, quality control of pharmaceutical raw materials, and registration of pharmaceutical products. In this study, we scrutinized the taxonomy of ‘Mahat’ by detailed examination of its morphology and distribution, including molecular and qualitative phytochemical studies. Leaf surfaces were inspected using scanning electron microscopy. The phylogeny of both species was studied using DNA sequences of nuclear and plastid regions. Chromatographic fingerprints, focusing on the major active compound oxyresveratrol, were identified using high-performance liquid chromatography. According to our current study, phylogenetic evidence showed that some samples of both species were clustered together in the same clade and phytochemical fingerprints were almost identical. These results are valuable data for taxonomic revision in the near future and reveal the possible utilization of *A. thailandicus* as a new material source of oxyresveratrol in the pharmaceutical industry.

## 1. Introduction

Moraceae is one of the most well-known plant families of the world, comprising about forty genera and over a thousand species [1]. The family is mainly distributed in the tropics [2]. Molecular and morphological evidences [3,4,5] indicate that the family is monophyletic; however, the classification at the tribal or infra-tribal level is still obscure due to the extraordinary diversity of complex inflorescence structures, pollination syndromes, and breeding systems [2]. The genus *Artocarpus* J.R. Forst. & G. Forst. is the third-largest genus of the family and the largest genus in the tribe Artocarpeae [2]. The center of distribution of the genus is in Borneo [6]. In 1847, Trécul divided the genus into two subgenera, *Jaca* Trécul and *Pseudojaca* Trécul [7]. The former subgenus has spirally arranged leaves, amplexicaul stipules, and annulate stipule scars, whereas the latter has alternate-distichous leaves, nonamplexicaul stipules, and lateral stipule scars. In 1959, Jarrett renamed the subgenus *Jaca* to *Artocarpus* [8,9]. Thereafter, Zerega and colleagues reduced the genus *Prainea* King to *Artocarpus* subgen. *Prainea* (King) Zerega, and the series *Cauliflori* Jarrett raised to *Artocarpus* subgen. *Cauliflori* (Jarrett) Zerega [2]. Thus, *Artocarpus* includes four subgenera, *Artocarpus*, *Pseudojaca*, *Prainea*, and *Cauliflori* [2].

In Thailand, the genus *Artocarpus* contains fourteen species with one endemic species, *A. thailandicus* C.C. Berg [10,11]. Several species are used for medicinal purposes, including *A. heterophyllus* Lam., *A. lacucha* Roxb. ex Buch.-Ham., *A. thailandicus*, and *A. altilis* (Parkinson) Fosberg. In Thai traditional medicine, the plant with the vernacular name ‘Mahat’ (common name: monkey jack) is widely used as an anthelmintic drug [12]. Furthermore, ‘Mahat’ is a major source of oxyresveratrol (*trans*-2,3′,4,5′-tetrahydroxystilbene), which is an active ingredient in skin whitening products, with antityrosinase, antioxidant, and anti-inflammatory activity [13,14,15,16,17]. Due to the morphological similarities, *A. lacucha* and *A. thailandicus* share the vernacular name ‘Mahat’. Although the Moraceae has already been revised in Flora of Thailand [11] with a great deal of taxonomic information, the key to species of the genus *Artocarpus* is based mainly on leaf and inflorescence characteristics which remain troublesome for the identification of ‘Mahat’. For example, presence of indumentum in the areoles are not constant characteristics in all examined specimens, or even in all leaves of the same specimen. This variation was also mentioned in Flora of Thailand [11]. Furthermore, the use of inflorescence characters can be problematic in non-flowering collections. This is affecting quality control of the raw materials in the pharmaceutical industry and the registration of pharmaceutical products with the Thai Food and Drug Administration (Thai FDA).

In this study, we attempted to verify the taxonomy of *A. lacucha* and *A. thailandicus*, which are used as the crude drug named ‘Mahat’ in Thai traditional medicine and herbal pharmaceutical products, using macroscopic and microscopic morphology, molecular phylogeny, and qualitative phytochemistry. We believed that our results provide valuable data for the taxonomic revision of ‘Mahat’ in the future as well as being helpful in the pharmaceutical product industry, as *A. thailandicus* is possibly a new source of plant raw material for oxyresveratrol consumption.

## 2. Results

### 2.1. Macroscopic Morphology and Distribution

**Figure 1:**
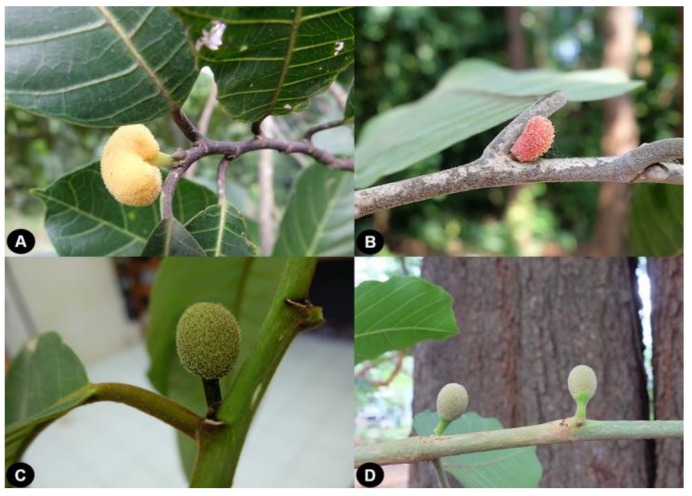
Inflorescences of *A. lacucha* and *A. thailandicus*. (**A**) staminate inflorescence of *A. lacucha*, subglobose (**B**) staminate inflorescence of *A. thailandicus*, obovoid (**C**) pistillate inflorescence of *A. lacucha*, obovoid (**D**) pistillate inflorescence of *A. thailandicus*, obovoid.

**Figure 2:**
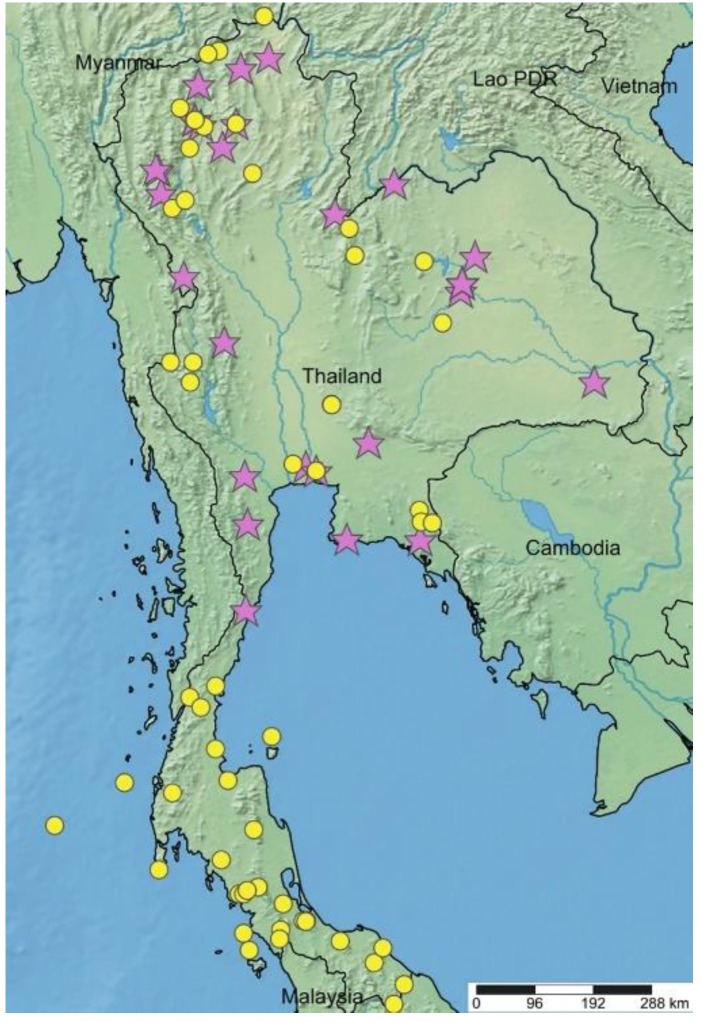
Distribution map of *A. lacucha* (yellow-circle) and *A. thailandicus* (pink-star) in Thailand.

The macroscopic morphology of collected samples and herbarium specimens were examined. The morphological characters of both species conformed to the plant description in Flora of Thailand [11]. The main characters differentiating the species are summarized in Figure 1 and Table 1. The distribution of both species in Thailand is presented in Figure 2. Furthermore, the typification details of *A. lacucha* were not mentioned in Flora of Thailand. However, during our herbarium observations we found the type specimen as shown below.

Type:—BANGLADESH. Rangpur: Chilmari, 16 December 1808, Collector Unknown 4655.B [holotype K (http://specimens.kew.org/herbarium/K001039600, male, and http://specimens.kew.org/herbarium/K001039601, female, last acc. 1 September 2018)].

In addition, there was the confusion of the nomenclature, i.e., *A. lacucha* and *A. lakoocha* Roxb., and its typification. Mabberley confirmed that *A. lacucha* was the correct scientific name and the type specimen was collected in K-W with Hamilton’s label [18]. The species name *A. lakoocha*, however, was mentioned in the samples’ DNA sequences obtained from GenBank database and it still was used in phylogenetic analyses in order to show that they are GenBank sequences. Moreover, in the plant description of *A. lacucha*, *A. dadah* Miq. was presented as one of the synonyms. Williams et al., in their phylogenetic and biogeographic investigation of *Artocarpus* showed that *A. dadah* showed monophyly with *A. lacucha* [6]. Hence, *A. dadah* should be discarded from the description in Flora of Thailand [11].

### 2.2. Leaf Surface Morphology

**Figure 3:**
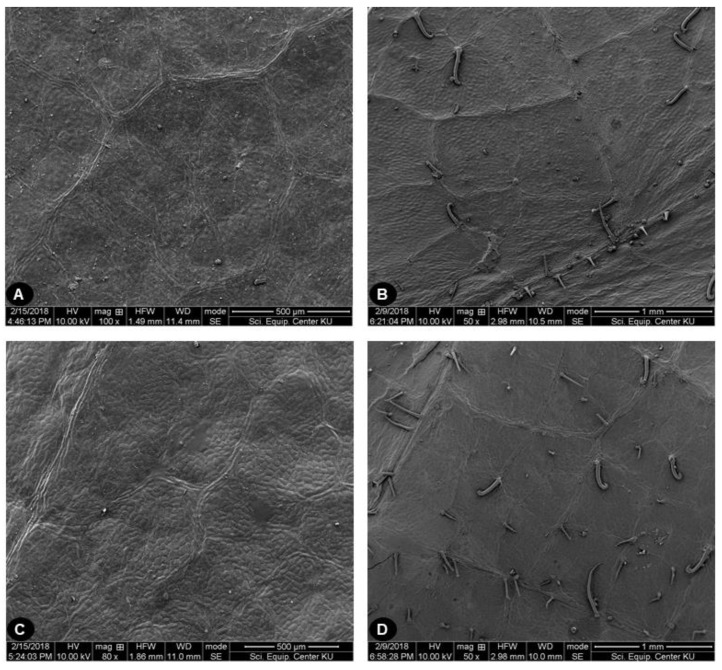
Upper surface of leaf micromorphology study by SEM of *A. lacucha* and *A. thailandicus*. (**A**) Upper surface of *A. lacucha* (voucher PBM05214), glabrous (**B**) Upper surface of *A. lacucha* (voucher PBM05218), with straight and uncinate smooth hair (**C**) Upper surface of *A. thailandicus*, glabrous (voucher PBM05203) (**D**) Upper surface of *A. thailandicus* (voucher PBM05229), with straight and uncinate smooth hair.

**Figure 4:**
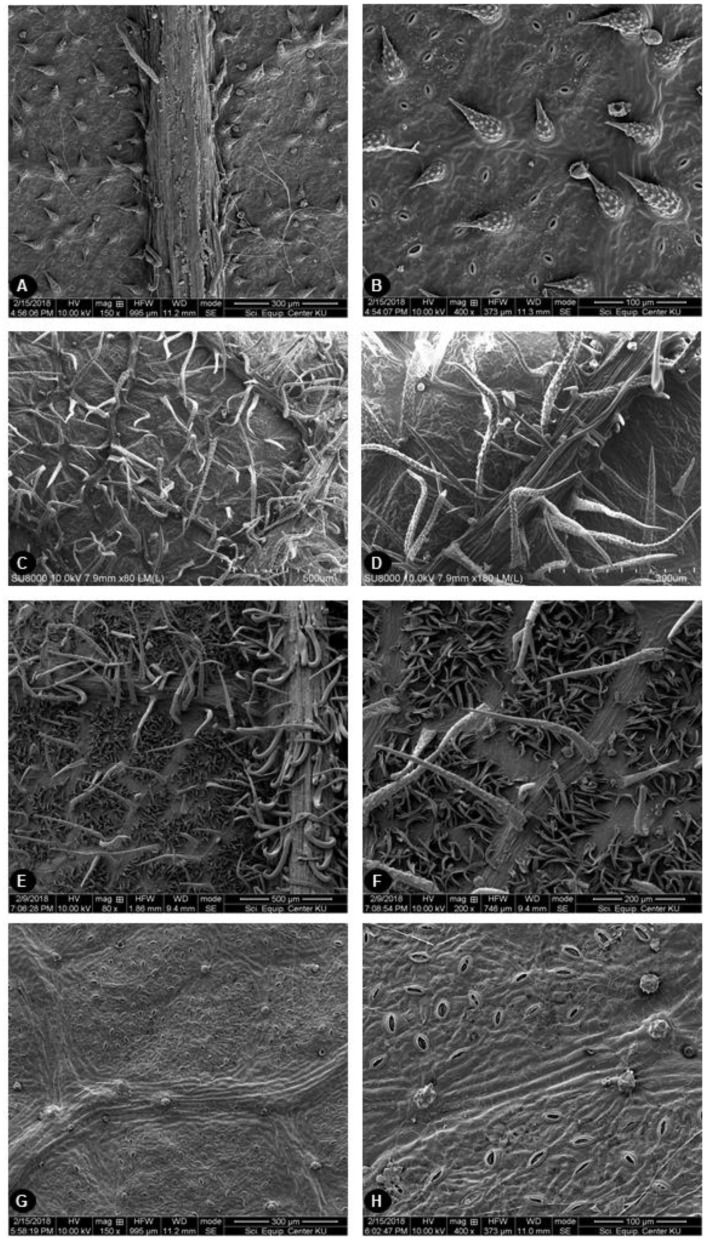
Lower surface of leaf micromorphology study by SEM of *A. lacucha* and *A. thailandicus*. (**A**,**B**) Lower surface of *A. lacucha* (PBM05214), gnarled papillae on the veinlets, sparsely smooth straight or uncinate hairs on lateral veins. (**C**,**D**) Lower surface of *A. lacucha* (PBM05225), gnarled long straight hairs mostly on veinlets. (**E**,**F**) Lower surface of *A. thailandicus* (PBM05229), gnarled long straight hairs mostly on the veinlets with tomentose hair in the areoles and densely smooth straight or uncinate hairs on the lateral veins. (**G**,**H**) Lower surface of *A. thailandicus* (PBM05205), gnarled papillae on the veinlets, lack of tomentose hair in the areoles.

Fourteen samples were selected, as representatives of the species and covering all floristic regions in Thailand, for scanning electron microscopy (SEM) of leaf surface characteristics. The upper and lower leaf surfaces, including the indumentum of individual species, are shown in Figure 3 and Figure 4, respectively. The upper leaf surfaces of both species were similar. They could be glabrous (Figure 3A,C) or have some straight and uncinate smooth hairs on the veins and lamina (Figure 3B,D). The lower leaf surfaces of specimens identified as *A. lacucha* had rather varied indumentum, from gnarled papillae to dense long straight-gnarled hairs on the veinlets, with smooth straight and/or uncinate hairs located sparsely on the lateral veins, but with almost glabrous areoles (Figure 4A–D). Similar to *A. lacucha*, the lower leaf surface of specimens identified as *A. thailandicus* had long straight-gnarled hairs on the veinlets. However, the areoles were densely covered with tomentose hairs and dense, smooth, straight, or uncinate hairs on the lateral veins (Figure 4E,F). In addition, lower surface of some *A. thailandicus* specimens (PBM05203, PBM05204, and PBM05205) showed gnarled papillae on the veinlets and lack of the indumentum in the areoles (Figure 4G,H).

### 2.3. Phylogenetics

**Figure 5:**
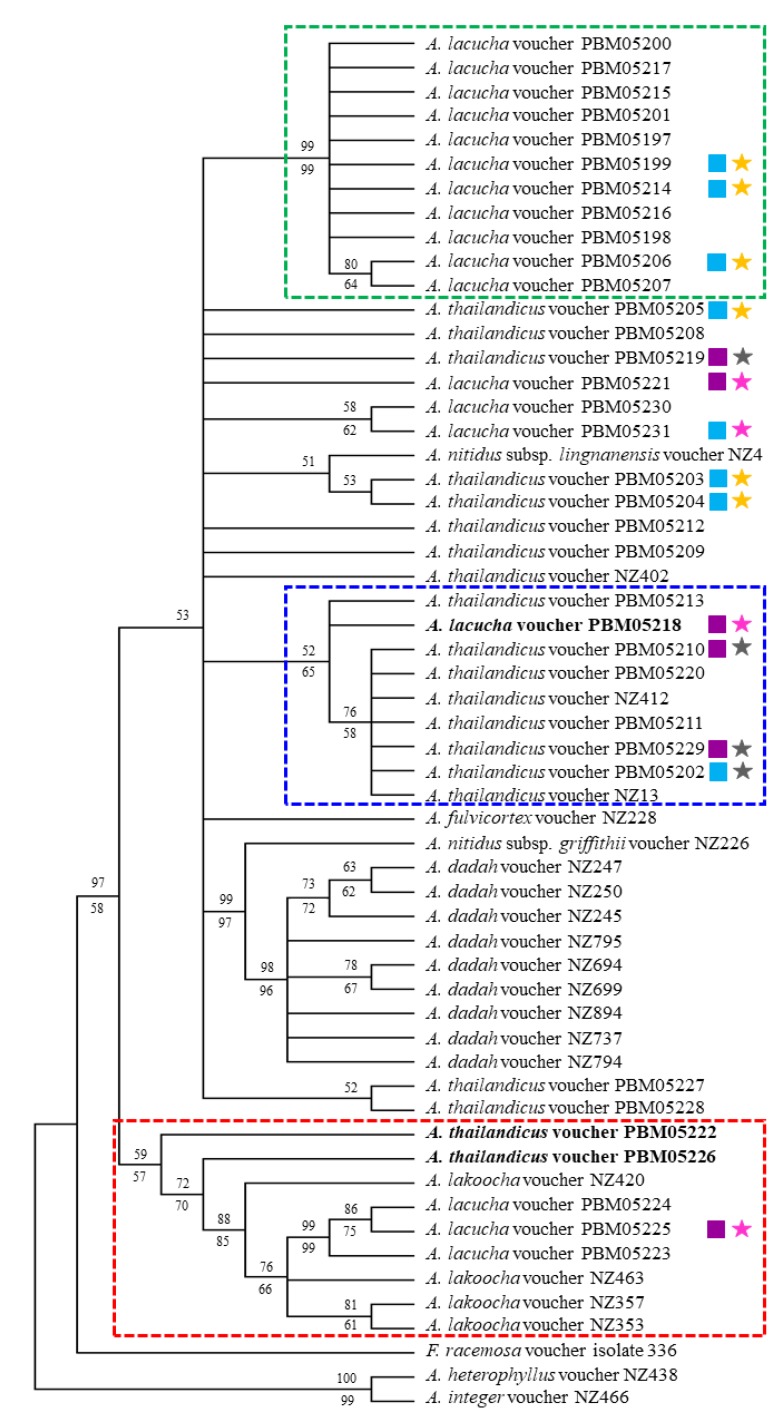
Maximum parsimony (MP) and maximum likelihood (ML) analysis of *A. lacucha* and *A. thailandicus* with other *Artocarpus* species. *A. integer* and *A. heterophyllus* were chosen as outgroup. The bootstrap percentage of MP and ML were shown as above and below the branches, respectively. Two minor clades, the blue and red square dashed line, indicated that both species were clustered together in the same clade with low bootstrap support. Some parts of the trees showed unresolved topology. Some samples of *A. lacucha* formed only one well-supported clade with 99% bootstrap support (indicated by green square dashed line). The SEM results were correlated. Light-blue square: upper surface glabrous; purple square: upper surface with straight and uncinate smooth hair; yellow star: lower surface with gnarled papillae on the veinlets, sparsely smooth straight or uncinate hairs on lateral veins; pink star: lower surface with gnarled long straight hairs mostly on veinlets; grey star: lower surface with gnarled long straight hairs mostly on the veinlets with tomentose hair in the areoles and densely smooth straight or uncinate hairs on the lateral veins.

Fifty-seven taxa were included in the phylogenetic analyses. All taxa provided ITS and *trn*H-*psb*A data, and thirty-five and fifty-six taxa provided ETS and *trn*L-F sequences, respectively. Maximum parsimony (MP) and maximum likelihood (ML) analyses of the separate markers, nuclear (ITS and ETS) and chloroplast (*trn*L-F and *trn*H-*psb*A) loci (Figures S1–S24) did not show any major conflicts in topology and thus all were combined (Figures S25–S28). The combined four aligned regions were 1726–1829 bp long. MP analysis of the four combined regions showed 741 potentially informative characters and resulted in three most parsimonious trees (MPTs) with a length = 282, the consistency index = 0.707, the retention index = 0.903 and the composite index = 0.779 (0.639) for all sites and parsimony-informative sites (in parentheses). For ML analysis, the discrete gamma distribution (5 categories (+G, parameter)) was 0.454. MP analysis conformed to ML analysis. The MP and ML consensus tree (Figure 5) revealed a major clade containing specimens of *A. lacucha*, *A. thailandicus* and some other *Artocarpus* species included in the analyses (sequences obtained from GenBank) with several minor clades. Most of them have low supports and some parts of the cladogram had an unresolved topology. Two minor clades, indicated in the blue and red square dashed line, showed that *A. lacucha* and *A. thailandicus* clustered together, with low bootstrap support. Both species were likely to be polyphyletic as they presented poor resolution of relationships. Only some samples of *A. lacucha* formed only one well-supported clade with 99% bootstrap support (indicated by green square dashed line). Likewise, *A. thailandicus* seems to be presented in more than two clades, all with low support values. Additionally, the leaf indumentum from SEM results were mapped and showed some correlation with cladogram, as shown in Figure 5.

### 2.4. Qualitative Phytochemistry

**Figure 6:**
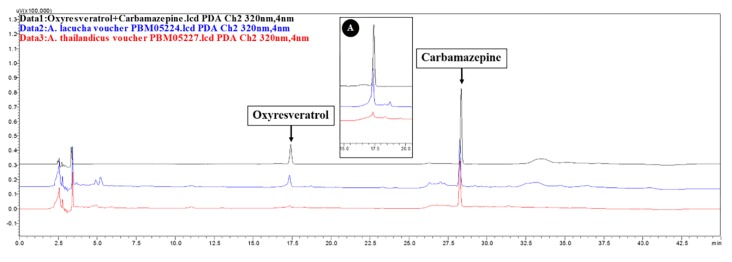
HPLC chromatograms of *A. lacucha* and *A. thailandicus* twig extract alignment. Oxyresveratrol standard and carbamazepine (internal standard) were detected at the retention time of 17.3 (Figure 6A) and 28.3 min, respectively. The HPLC chromatograms of oxyresveratrol (black line), *A. lacucha* voucher PBM05224 (blue line) and *A. thailandicus* voucher PBM05227 (red line) were selected as representative of each species and aligned in order to comparison.

In this study, we focused mainly on the qualitative analysis of the major active compound, oxyresveratrol, in the extract of both species by high-performance liquid chromatography (HPLC). Based on the HPLC chromatograms produced by both species were almost identical (Figure 6). Oxyresveratrol was detected at the retention time of 17.3 min in the extract of both species.

## 3. Discussion

There have been many publications reporting the use of ‘Mahat’ from Thailand as a material source in various fields such as biogeography, phylogeny, phytochemistry, and biological activity testing. Nevertheless, *A. lacucha* [6,12,15,16,19,20,21,22,23] has been cited more frequently than *A. thailandicus* [6,24]. This is likely because the name *A. lacucha* is better-known and has been used for longer time (since 1814) [25] than *A. thailandicus* (since 2005) [10]. The confusion over the naming of these plants has been exacerbated by the high variation in their vegetative characters. Furthermore, the heartwood, which has no distinguishing characteristics, has also been used for research and pharmaceutical production. It is thus more difficult to distinguish the species origin of ‘Mahat’ raw materials, and consequently their adulteration. Therefore, further taxonomic investigation of these two species in Thailand is needed. If there are other evidences that reject the monophyly of *A. thailandicus*, its taxonomic status at species level should also be discarded. According to our studies, however, samples of *A. lacucha* were collected only in Thailand, while this species is distributed from South, East and Southeast Asia to Oceania [11]. More samples of *A. lacucha* from the areas outside Thailand should be included into the analyses to clarify the complexity of both species.

The species circumscriptions of *Artocarpus* subgen. *Pseudojaca* is one of the most difficult [6]. The alteration of taxonomic rank at species level could occur due to variations in leaf and inflorescence morphology [6]. Although the morphology of *A. lacucha* and *A. thailandicus* are different and appear sufficient to be used for efficient identification, there are some limitations to those characters. According to the taxonomic key of the genus *Artocarpus* in Flora of Thailand [11] and the results from our study, the presence of hairs in the areoles may be used for the identification of *A. thailandicus*. This character was dominant, particularly when examined under a stereo microscope, but the variation in hair density was observed in some specimens. Nevertheless, in some examined specimens, this character was absent (*Pooma et al. 5749*, *Aneklaphakij 10*, *Aneklaphakij 11*, *Aneklaphakij 13*). The specimen “*Wongprasert s.n.*” showed a presence of hairs only in some leaves. Additionally, this variation was also mentioned in Flora of Thailand [11]. Moreover, the inflorescence is another significant part used to differentiate each species and can be problematic in non-flowering specimens. Furthermore, we hypothesized that Berg et al. [11] examined mostly the ancient herbaria specimens using only stereo microscope with less magnification than SEM. Thus, some characteristics could not be observed; for example, the gnarled papillae on the veinlets could be seen only under SEM. Therefore, our leaf morphology result was probably different from the previous work [11] and could imply that the species identification using leaf indumentum might not be suitable criterion, although, the characteristic is not affected by environmental factors (the first author’s personal observation). The second character was indumentum color, which is white and brown, the interpretation of which may be subjective. The third character was the length of the peduncle of staminate and pistillate inflorescences, with *A. lacucha* having longer peduncles than *A. thailandicus*. However, this character could vary during different stages of maturity. Moreover, it is suggested that hybridization between these two species is likely to occur [11]. Evidences of interspecific hybridization are still unclear and hybrid determination is required. However, this biological phenomenon can be common in the genus *Artocarpus*, as found among species of commercial breadfruits, *A. altilis* (Park.) Fosberg, *A. mariannensis* Trécul and *A. camansi* Blanco, whose hybrids shared morphological and cytological characters [26]. Cross-pollination by wind with the small, powdery pollen grains spread were also reported for some species of breadfruit trees [27,28]. Therefore, it can be hypothesized that interspecific hybridization between *A. lacucha* and *A. thailandicus* can also be possible. The MP & ML strict consensus trees showed that some of *A. lacucha* and *A. thailandicus* occurred in the same clade, with low bootstrap support, and tree topology was unresolved. This phenomenon could occur when studying the population scale as found in in the genus *Secale* L. (Poaceae) by Maraci and colleagues [29]. It may be caused by the high similarity in the sequences of both species and the other *Artocarpus* spp. included in the study or the speciation of them might be based on other evidences rather than molecular data such as geographical origin [29]. Even though both species evolved from the same common ancestor, as in the Williams and colleagues’ study [6], the incongruence between morphological characters and molecular evidence, which is a common phenomenon in Moraceae such as *Ficus krishnae* C. DC. [30], could result from asymmetric evolution rates in DNA sequences and phenotypic morphogenesis. In addition, from this phenomenon, the connected morphological variation and molecular data probably could not be observed. Nevertheless, there was a well-defined clade (indicated by green square dashed line, Figure 5) of *A. lacucha*. We discovered that the SEM results of samples PBM05199, PBM05206, and PBM05214 shared the synapomorphic characters which the upper leaf surface was glabrous and lower leaf surface was gnarled papillae on the veinlets, sparsely smooth straight or uncinate hairs on lateral veins. Therefore, we attempted to correlate the micromorphology of leaf surface with molecular data as shown in Figure 5. Only a specimen in the early diverging clade (encircled by red square dashed line) was SEM analyzed and it shows straight and uncinate smooth hair on upper surface and gnarled long straight hairs mostly on veinlets of leaf lower surface. Those characteristics of leaf indumentum were hypothesized to be also present among other samples in this clade and also conformed to the description in Flora of Thailand [11], even though Berg et al. did not use SEM for leaf morphology examination. However, those features have evolved more than once in phylogeny of ‘Mahat’, as shown in Figure 5. For the later diverging clades (encircled by green and blue square dashed line), they could be noticed that later evolved three traits: 1. glabrous upper surface (shown by light blue square), 2. gnarled papillae on the veinlets with sparsely smooth straight or uncinate hairs on lateral veins (shown by yellow star), and 3. gnarled long straight hairs mostly on the veinlets with tomentose hair in the areoles and densely smooth straight or uncinate hairs on the lateral veins on lower surface (shown by grey star). Leaf indumentum is beneficial for plant protection against herbivory, pathogens or strong sunlight and also relates to drought tolerance by facilitating condensation of air moisture onto the plant surface [31,32,33]. The upper surface of *A. lacucha*, however, seemingly become more glabrous and the reasons of this evolutionary trend are still unclear. In order to compensate the absence of leaf indumentum on the upper surface, *A. lacucha* might develop a thicker cuticle. More detailed studies of leaf anatomy, especially the cuticle thickness, are required to understand trend of evolution in *A. lacucha*’s leaf indumentum. Moreover, leaf anatomical characteristics apart from indumentum are potentially useful for taxonomic purposes in Moraceae. For example, in *Ficus* subsection *Urostigma*, the epidermis and lithocysts characteristics could be used for identification of *Ficus* subsection *Conosycea* [34]. Since the relationship between these two species are still not well-supported to ensure species circumscription, this result needs to be confirmed. Sampling from a wider range of distribution, including areas outside Thailand and the use of additional DNA markers, might help to resolve the taxonomy of ‘Mahat’. Most of the morphological differences observed are relevant to the expression of important regulatory genes [35,36]; however, the studied DNA regions might be housekeeping regions that are not involve in the expression of the relevant characters or not involved in their regulation [30]. Thus, genes related to the expression of morphological characters that differ between these two species should be investigated in future.

Chromatography is one tool for classifying and identifying plants, especially cases where plants produce unique chromatogram patterns for specific chemical markers [37,38]. We investigated the use of high-performance liquid chromatography to differentiate the phytochemical fingerprint of the major active compound, oxyresveratrol, in the extract of both species. Oxyresveratrol is commonly collected from the heartwood; however, we tested twigs in this study due to the limitations of sample collection. Oxyresveratrol was detected in both species’ extracts; however, there is likely variation caused by differences in season, age and geography of the samples. The aligned chromatograms showed high similarity among species. Thus, phytochemical analysis conformed to the molecular studies, that the species could not be differentiated. Moreover, these species can be used interchangeably in traditional medicine and pharmaceutical products as the active compound is identical. However, future studies should investigate metabolites using high-performance liquid chromatography coupled with high-resolution mass spectrometry.

According to our study, both molecular technique and phytochemical study focusing on major active compound could be useful tools for discrimination of plant species, especially in the quality control of raw materials for pharmaceutical product production. Generally, the raw material is in a powder form which is very difficult for plant species identification. Therefore, the molecular technique and qualitative phytochemical analysis can be used for raw material quality examination prior using for production. These two techniques are very suitable because a small amount of sample is used, the process consumes a moderate amount of time and could reduce production costs in cases where undesired raw material is found before mass production.

## 4. Materials and Methods

### 4.1. Taxonomic Study

The plant samples were collected from the fieldwork between October 2015 and November 2017. For representativeness, plants from all Thai floristic regions were sampled [11]. All the samples were first identified using the identification key presented in Flora of Thailand. Each sample was examined under the stereo and light microscopes supplemented with an ocular micrometer. All voucher specimens were deposited at the Herbarium of Department of Pharmaceutical Botany (PBM), Mahidol University, Thailand. There were 35 collections in this study. In total, 18 samples were identified to *A. lacucha*, collected from Northern, North-eastern, Central and Peninsular regions of Thailand, whereas 17 samples identified as *A. thailandicus* were collected from all floristic regions except Peninsular region. The list of studied samples is shown in Table 2. Moreover, 72 and 23 specimens of *A. lacucha* and *A. thailandicus*, respectively, from BK, BKF, E, K, L, and PBM (Thiers 2019 [continuously updated]) were also studied. All specimens were seen by the first author. The GPS coordinates of the collections were retrieved from the herbarium labels, the fuzzy gazetteer (http://isodp.hof-university.de/fuzzyg/query) and Google Maps. The map of distribution in Thailand based on the localities of all collections was constructed using SimpleMappr, http://www.simplemappr.net/ [39].

### 4.2. Scanning Electron Microscopy (SEM)

Leaves were collected at the third to the fourth node from the branch apex, with at least three leaves for each accession. Samples were cleaned and dried in a hot air oven at 60 °C for a day, then preserved in silica gel for the subsequent scanning electron microscope (SEM) study. The upper and lower leaf surfaces of seven samples of *A. lacucha* (voucher PBM05199, PBM05206, PBM05214, PBM05218, PBM05221, PBM05225, PBM05231) and *A. thailandicus* (voucher PBM05202, PBM05203, PBM05204, PBM05205, PBM05210, PBM05219, PBM05229) were selected as representatives of the species and all Thai floristic regions. Dried leaves were cut to the size of 5 × 5 mm from the median area between the margin and midrib, and mounted directly on stubs using double-sided carbon tape, then coated with gold by a Polaron Range SC7620 Sputter Coater & CA7625 Carbon Accessary and platinum palladium by Hitachi E102 sputter coater, Japan. The samples were photographed by FEI Quanta 450, Hillsboro, OR, USA (accelerating voltage at 10.0 kV) at the Scientific Equipment Center, Faculty of Science, Kasetsart University and by Hitachi SU8010, Japan (accelerating voltage at 10.0 kV) at the Center of Nanoimaging, Faculty of Science, Mahidol University, Bangkok, Thailand.

### 4.3. DNA Extraction, Amplification and Sequencing

Young leaves of collected samples were dried and stored in silica gel for DNA extraction. They were milled into the fine powder using a mortar and pestle. The DNA extraction process followed the protocol of the plant genomic DNA extraction kit (RBC Bioscience, Taiwan). Purified DNA was kept at −20 °C before being used as a template in a polymerase chain reaction (PCR). In this study, specific primer sequences in Table 3 (synthesized by Macrogen Inc., Seoul, South Korea) were employed for amplifying the nuclear genome (ITS and ETS region) and chloroplast genome (*trn*L-F and *trn*H-*psb*A intergenic spacer). PCR buffer, dNTPs and Taq polymerase reagents were purchased from RBC Bioscience, Taiwan. DNase/RNase free distilled water was bought from Invitrogen, USA. The PCR condition of each gene was based on previous studies (See in the reference of each primer sequences). After the PCR reaction, the product was checked using gel electrophoresis. The PCR products were purified using a HiYield™ Gel/PCR DNA Fragments Extraction Kit, then the concentration was determined using a SmartSpec^TM^ Plus Spectrophotometer (Bio-rad, Philadelphia, PA, USA). The samples containing a concentration of more than 50 ng/µL were delivered for sequencing. For the sequencing procedure, the capillary electrophoresis sequencing method (standard sequencing) was performed by Macrogen sequencing services (Macrogen Inc., Seoul, South Korea). Apart from our samples, we retrieved four samples of *A. lacucha* and three samples of *A. thailandicus* from the NCBI GenBank database to be included in the phylogenetic analysis. However, these samples contain only ITS region, *trn*L-F and *trn*H-*psb*A intergenic spacer.

### 4.4. Sequence Alignment and Phylogenetic Analysis

Subsequent to the sequencing process being accomplished, each sequence was aligned by ClustalW [44] using the biological sequence alignment editor (BioEdit). The positions containing more than 75% missing or undetermined characters were eliminated. The analyses of each separate locus, nuclear and chloroplast loci, as well as four combined molecular DNA markers were analyzed using the maximum parsimony (MP) and maximum likelihood (ML) method for phylogenetic tree reconstruction by molecular evolutionary genetics analysis version 7.0 (MEGA7) [45]. The MP tree was obtained using the Tree-Bisection-Regrafting (TBR) algorithm [46] (p. 126) with search level 1 in which the initial trees were obtained by the random addition of sequences (10 replicates). Model selection for ML analysis was conducted using the model selection tool as implemented in MEGA7. From the model test result, the ML tree was conducted based on the Tamura 3-parameter model [47] for four combined regions; chloroplast loci; *trn*L-F and *trn*H-*psb*A intergenic spacer, while Kimura 2-parameter model [48] was used for the nuclear loci, ITS, and ETS regions. The initial tree for the heuristic search was obtained automatically by applying Neighbor-Join and BioNJ algorithms to a matrix of pairwise distances estimated using the Maximum Composite Likelihood (MCL) approach and then selecting the topology with a superior log-likelihood value. For four combined regions and nuclear loci, a discrete gamma distribution was used to model evolutionary rate differences among the sites (5 categories (+G, parameter)). In both methods, the bootstrap strict consensus tree inferred from 1000 replicates was taken to represent the evolutionary history of the taxa analyzed and the percentage of replicate trees in which the associated taxa clustered together in the bootstrap test (1000 replicates) were shown next to the branches [49]. Branches corresponding to partitions reproduced in less than 50% bootstrap replicates were collapsed. The sequences of each region from each sample were submitted into the NCBI GenBank database. All of the accession numbers were shown in Table 4. The sequences of *A. lacucha*, *A. thailandicus*, *A. dadah*, *A. heterophyllus* Lam., *A. nitidus* subsp. *griffithii* (King) F.M. Jarrett, *A. nitidus* subsp. *lingnanensis* (Merr.) F.M. Jarrett, *A. fulvicortex* F.M. Jarrett, *A. integer* (Thunb.) Merr., and *Ficus racemosa* L. in the NCBI GenBank database were retrieved and also included in the phylogenetic analysis. In addition, *A. integer* and *A. heterophyllus* were selected as outgroup. Bootstrap percentages were described by Chantarasuwan and colleagues [40] as strong (85–100%), moderate (75–84%), low (50–74%), and no support (≤50%).

### 4.5. Qualitative Phytochemical Analysis

The twigs of all collected samples were chopped, dried in a hot air oven at 60 °C for 1–2 days and ground using a hammer miller into the moderated powder (through sieve No.14), then kept in a dry place. The high-performance liquid chromatographic (HPLC) method was used for qualitative phytochemical analysis. The twig powder of each sample was macerated with methanol AR grade (Fisher Scientific, Pittsburgh, PA, USA) in the ratio of 1:30 at room temperature for 72 h on the shaker (100 rpm). The extract was filtered by Whatman No.1 filter paper, followed by evaporating to dryness under reduced pressure at 40 °C using a rotary evaporator (Buchi, Flawil, Switzerland). All of the extracts were diluted with methanol HPLC grade (Honeywell Burdick & Jackson™, Muskegon, MI, USA) to the concentration of 1 mg/mL before injection. The chromatographic analysis was performed on a Shimadzu, LC-20AD liquid chromatography system containing a quaternary solvent delivery system, autosampler (SIL-20AHT), degasser (DGU-20A5R), column oven (CTO-20A) and photodiode array detector (PAD, SPD-M20A). Each chromatographic data acquisition was performed using Lab Solutions software. The completely validated chromatographic condition was adapted from the previous study [50]. Chromatographic separation was achieved on a C18 reverse phase column (Phenomenex, Torrance, CA, USA, Gemini-NX C18, 250 mm × 4.6 mm i.d., 5 µm) using a 1 mL/min mobile phase gradient system. The mobile phase comprises acetonitrile (Sigma, St. Louis, MO, USA) and 0.5% aqueous acetic acid (*v*/*v*). The gradient elution profile was 18%–25% acetonitrile at 0–10 min, and 25%–40% acetonitrile at 10–25 min, which was then returned to equilibration at the initial conditions for 10 minutes before the injection of the next sample. The column temperature was maintained at 30 °C and the injection volume was 20 µL. The detection was examined at 320 nm. The oxyresveratrol standard was prepared at a concentration of 6.25 µg/mL and injected with the same amount and under the same conditions as the sample. Carbamazepine (Himedia, Mumbai, India) in the concentration of 100 µg/mL was augmented as the internal standard.

## 5. Conclusions

‘Mahat’ is a well-known medicinal plant in Thailand. The ambiguity of the taxonomic classification of the source of ‘Mahat’ is pivotal for quality control of its pharmaceutical product yields. We revised the plant morphology and distribution and investigated the molecular evidence and phytochemical fingerprints. Morphological characters were highly variable especially the indumentum in the areoles, and phylogenetic evidence demonstrated that *A. lacucha* is likely to be polyphyletic, with only one well-supported clade. In addition, *A. thailandicus* seems to form more than one clade. Moreover, qualitative phytochemical analysis by HPLC showed that the phytochemical fingerprints of the major active compound, oxyresveratrol, were almost identical. Hence, *A. thailandicus* might be utilized in pharmaceutical industry as another new plant material source of oxyresveratrol in the future. According to our present results, we still keep both *A. lacucha* and *A. thailandicus* as separate species even if the molecular and phytochemical evidences show these two species could be the same. In order to reduce *A. thailandicus* as a synonym of *A. lacucha*, further deeply detailed taxonomic researches of both species should be re-investigated.

## Figures and Tables

**Table 1:** The main differentiating characters between *A. lacucha* and *A. thailandicus*.

Characters	*A. lacucha*	*A. thailandicus*
Indumentum of the areoles on the lower surface of lamina	Absent	Present
Shape and size of staminate inflorescence	Mostly subglobose 0.6–2 cm diam.	Mostly obovoid, cylindrical, or clavate 0.2–0.4 cm diam.
Size of pistillate inflorescence	1.5 cm diam.	0.7–1 cm diam.
The length of peduncle of staminate inflorescence	0.2–2 cm	Sessile or up to 0.1 cm
The length of peduncle of pistillate inflorescence	0.2–4 cm	Up to 0.7 cm

**Table 2:** List of the studied materials.

Taxon	Floristic Region	Locality	PBM Number	Collector Number
*A. lacucha*	Central	Saraburi, Chaloem Phra Kiat, lat 14.67633° N lon 100.8868° E	05197	*Aneklaphakij 2*
	Central	Saraburi, Chaloem Phra Kiat, lat 14.67217° N lon 100.887° E	05198	*Aneklaphakij 4*
	Central	Saraburi, Chaloem Phra Kiat, lat 14.66953° N lon 100.8898° E	05199	*Aneklaphakij 5*
	Central	Nakhon Pathom, Phutthamonthon, lat 13.79072° N lon 100.317° E	05200	*Aneklaphakij 6*
	Central	Nakhon Pathom, Phutthamonthon, lat 13.79072° N lon 100.317° E	05201	*Aneklaphakij 7*
	North-eastern	Khon Kaen, Wiang Kao, lat 16.79369° N lon 102.2577° E	05206	*Aneklaphakij 14*
	North-eastern	Khon Kaen, Wiang Kao, lat 16.79369° N lon 102.2577° E	05207	*Aneklaphakij 15*
	North-eastern	Khon Kaen, Phon, lat 15.88319° N lon 102.5332° E	05214	*Aneklaphakij 23*
	North-eastern	Khon Kaen, Phon, lat 15.88319° N lon 102.5332° E	05215	*Aneklaphakij 24*
	North-eastern	Khon Kaen, Phon, lat 15.88319° N lon 102.5332° E	05216	*Aneklaphakij 25*
	North-eastern	Khon Kaen, Phon, lat 15.88319° N lon 102.5332° E	05217	*Aneklaphakij 26*
	North-eastern	Khon Kaen, Phon, lat 15.88319° N lon 102.5332° E	05218	*Aneklaphakij 27*
	Central	Bangkok, Prawet, lat 13.68917° N lon 100.6596° E	05221	*Aneklaphakij 31*
	Northern	Chiang Mai, Mae Rim, lat 18.89464° N lon 98.85864° E	05223	*Aneklaphakij 36*
	Northern	Chiang Mai, Mae Rim, lat 18.89456° N lon 98.85869° E	05224	*Aneklaphakij 37*
	Northern	Chiang Mai, Mae Rim, lat 18.89133° N lon 98.86214° E	05225	*Aneklaphakij* 38
	Peninsular	Songkhla, Hat Yai, lat 7.008417° N lon 100.5059° E	05230	*Aneklaphakij 44*
	Peninsular	Songkhla, Hat Yai, lat 7.008528° N lon 100.5062° E	05231	*Aneklaphakij 47*
*A. thailandicus*	South-western	Phetchaburi, Kaeng Krachan, lat 12.9025° N lon 99.64325° E	05202	*Aneklaphakij 9*
	Eastern	Ubon Ratchathani, Samrong, lat 15.01044° N lon 104.7846° E	05203	*Aneklaphakij 10*
	Eastern	Ubon Ratchathani, Samrong, lat 15.01044° N lon 104.7846° E	05204	*Aneklaphakij 11*
	South-eastern	Prachin Buri, Mueang Prachin Buri, lat 14.10967° N lon 101.4367° E	05205	*Aneklaphakij 13*
	North-eastern	Khon Kaen, Mueang Khon Kaen, lat 16.4695° N lon 102.8271° E	05208	*Aneklaphakij 17*
	North-eastern	Khon Kaen, Mueang Khon Kaen, lat 16.47194° N lon 102.8218° E	05209	*Aneklaphakij 18*
	North-eastern	Khon Kaen, Mueang Khon Kaen, lat 16.47706° N lon 102.8258° E	05210	*Aneklaphakij 19*
	North-eastern	Khon Kaen, Mueang Khon Kaen, lat 16.47706° N lon 102.8258° E	05211	*Aneklaphakij 20*
	North-eastern	Khon Kaen, Mueang Khon Kaen, lat 16.47975° N lon 102.8276° E	05212	*Aneklaphakij 21*
	North-eastern	Khon Kaen, Mueang Khon Kaen, lat 16.47447° N lon 102.8254° E	05213	*Aneklaphakij 22*
	Central	Bangkok, Prawet, lat 13.68853° N lon 100.6606° E	05219	*Aneklaphakij 28*
	Central	Bangkok, Prawet, lat 13.68853° N lon 100.6606° E	05220	*Aneklaphakij 30*
	Northern	Chiang Mai, Mae Rim, lat 18.89742° N lon 98.85928° E	05222	*Aneklaphakij 33*
	Northern	Chiang Mai, Mae Rim, lat 18.89136° N lon 98.8625° E	05226	*Aneklaphakij 39*
	Northern	Chiang Mai, Mae Rim, lat 18.88869° N lon 98.86169° E	05227	*Aneklaphakij 40*
	Northern	Chiang Mai, Mae Rim, lat 18.88869° N lon 98.86169° E	05228	*Aneklaphakij 41*
	Northern	Chiang Mai, Mae Rim, lat 18.89° N lon 98.85842° E	05229	*Aneklaphakij 42*

**Table 3:** Primer sequences used in the study.

Region	Primer Sequence	References
ITS	Forward: 5’–AACAAGGTTTCCGTAGGTGA–3’ Reverse: 5’–TATGCTTAAAYTCAGCGGGT–3’	[2]
ETS	Forward: 5’–GACCCTTGGTTCCTGTGTTGC–3’ Reverse: 5’–ACTTACACATGCATGGCTTAATCT–3’	[40,41]
*trn*L-F	primer c: 5’–CGAAATCGGTAGACGCTACG–3’ primer d: 5’–GGGGATAGAGGGACTTGAAC–3’ primer e: 5’–GGTTCAAGTCCCTCTATCCC–3’ primer f: 5’–ATTTGAACTGGTGACACGAG–3’	[42]
*trn*H-*psb*A	Forward: 5’–CGCGCATGGTGGATTCACAAATC–3’ Reverse: 5’–GTTATGCATGAACGTAATGCTC–3’	[43]

**Table 4:** Accession number of the sequences deposited in NCBI GenBank database.

PBM Number	ITS	ETS	*trn*L-F	*trn*H-*psb*A
05197	MK850217	MK873914	MK873937	MK873960
05198	MK850218	MK873915	MK873938	MK873961
05199	MH801139	MH807231	MH817052	MH817064
05200	MK850219	MK873916	MK873939	MK873962
05201	MK850220	MK873917	MK873940	MK873963
05202	MH801140	MH807232	MH817053	MH817065
05203	MH801141	MH807233	MH817054	MH817066
05204	MK850221	MK873918	MK873941	MK873964
05205	MH801142	MH807234	MH817055	MH817067
05206	MH801143	MH807235	MH817056	MH817068
05207	MK850222	MK873919	MK873942	MK873965
05208	MK850223	MK873920	MK873943	MK873966
05209	MK850224	MK873921	MK873944	MK873967
05210	MH801144	MH807236	MH817057	MH817069
05211	MK850225	MK873922	MK873945	MK873968
05212	MK850226	MK873923	MK873946	MK873969
05213	MK850227	MK873924	MK873947	MK873970
05214	MK850228	MK873925	MK873948	MK873971
05215	MK850229	MK873926	MK873949	MK873972
05216	MK850230	MK873927	MK873950	MK873973
05217	MK850231	MK873928	MK873951	MK873974
05218	MH801145	MH807237	MH817058	MH817070
05219	MH801146	MH807238	MH817059	MH817071
05220	MK850232	MK873929	MK873952	MK873975
05221	MH801147	MH807239	MH817060	MH817072
05222	MK850233	MK873930	MK873953	MK873976
05223	MK850234	MK873931	MK873954	MK873977
05224	MK850235	MK873932	MK873955	MK873978
05225	MH801148	MH807240	MH817061	MH817073
05226	MK850236	MK873933	MK873956	MK873979
05227	MK850237	MK873934	MK873957	MK873980
05228	MK850238	MK873935	MK873958	MK873981
05229	MH801149	MH807241	MH817062	MH817074
05230	MK850239	MK873936	MK873959	MK873982
05231	MH801150	MH807242	MH817063	MH817075

## Supplementary Materials

The following are available online at https://www.mdpi.com/2223-7747/9/3/391/s1, Figure S1: ITS region maximum parsimony consensus tree of *A. lacucha* and *A. thailandicus* with other *Artocarpus* species. *A. integer* and *A. heterophyllus* were chosen as an outgroup, Figure S2: ETS region maximum parsimony consensus tree of *A. lacucha* and *A. thailandicus*, Figure S3: *trn*L-F intergenic spacer maximum parsimony consensus tree of *A. lacucha* and *A. thailandicus* with other *Artocarpus* species. *A. integer* and *A. heterophyllus* were chosen as an outgroup, Figure S4: *trn*H-*psb*A intergenic spacer maximum parsimony consensus tree of *A. lacucha* and *A. thailandicus* with other *Artocarpus* species. *F. racemosa* was chosen as an outgroup, Figure S5: Nuclear loci maximum parsimony consensus tree of *A. lacucha* and *A. thailandicus* with other *Artocarpus* species. *A. integer* and *A. heterophyllus* were chosen as an outgroup, Figure S6: Chloroplast loci maximum parsimony consensus tree of *A. lacucha* and *A. thailandicus* with other *Artocarpus* species. *F. racemosa* was chosen as an outgroup, Figure S7: ITS region maximum likelihood consensus tree of *A. lacucha* and *A. thailandicus* with other *Artocarpus* species. *A. integer* and *A. heterophyllus* were chosen as an outgroup, Figure S8: ETS region maximum likelihood consensus tree of *A. lacucha* and *A. thailandicus*, Figure S9: *trn*L-F intergenic spacer maximum likelihood consensus tree of *A. lacucha* and *A. thailandicus* with other *Artocarpus* species. *A. integer* and *A. heterophyllus* were chosen as an outgroup, Figure S10: *trn*H-*psb*A intergenic spacer maximum likelihood consensus tree of *A. lacucha* and *A. thailandicus* with other *Artocarpus* species. *F. racemosa* was chosen as an outgroup, Figure S11: Nuclear loci maximum likelihood consensus tree of *A. lacucha* and *A. thailandicus* with other *Artocarpus* species. *A. integer* and *A. heterophyllus* were chosen as an outgroup, Figure S12: Chloroplast loci maximum likelihood consensus tree of *A. lacucha* and *A. thailandicus* with other *Artocarpus* species. *F. racemosa* was chosen as an outgroup, Figure S13: ITS region maximum parsimony (without branches collapsed) tree of *A. lacucha* and *A. thailandicus* with other *Artocarpus* species. *A. integer* and *A. heterophyllus* were chosen as an outgroup, Figure S14: ETS region maximum parsimony (without branches collapsed) tree of *A. lacucha* and *A. thailandicus*, Figure S15: *trn*L-F intergenic spacer maximum parsimony (without branches collapsed) tree of *A. lacucha* and *A. thailandicus* with other *Artocarpus* species. *A. integer* and *A. heterophyllus* were chosen as an outgroup, Figure S16: *trn*H-*psb*A intergenic spacer maximum parsimony (without branches collapsed) tree of *A. lacucha* and *A. thailandicus* with other *Artocarpus* species. *F. racemosa* was chosen as an outgroup, Figure S17: Nuclear loci maximum parsimony (without branches collapsed) tree of *A. lacucha* and *A. thailandicus* with other *Artocarpus* species. *A. integer* and *A. heterophyllus* were chosen as an outgroup, Figure S18: Chloroplast loci maximum parsimony (without branches collapsed) tree of *A. lacucha* and *A. thailandicus* with other *Artocarpus* species. *F. racemosa* was chosen as an outgroup, Figure S19: ITS region maximum likelihood (without branches collapsed) tree of *A. lacucha* and *A. thailandicus* with other *Artocarpus* species. *A. integer* and *A. heterophyllus* were chosen as an outgroup, Figure S20: ETS region maximum likelihood (without branches collapsed) tree of *A. lacucha* and *A. thailandicus*, Figure S21: *trn*L-F intergenic spacer maximum likelihood (without branches collapsed) tree of *A. lacucha* and *A. thailandicus* with other *Artocarpus* species. *A. integer* and *A. heterophyllus* were chosen as an outgroup, Figure S22: *trn*H-*psb*A intergenic spacer maximum likelihood (without branches collapsed) tree of *A. lacucha* and *A. thailandicus* with other *Artocarpus* species. *F. racemosa* was chosen as an outgroup, Figure S23: Nuclear loci maximum likelihood (without branches collapsed) tree of *A. lacucha* and *A. thailandicus* with other *Artocarpus* species. *A. integer* and *A. heterophyllus* were chosen as an outgroup, Figure S24: Chloroplast loci maximum likelihood (without branches collapsed) tree of *A. lacucha* and *A. thailandicus* with other *Artocarpus* species. *F. racemosa* was chosen as an outgroup, Figure S25: Four combined regions maximum parsimony consensus tree of *A. lacucha* and *A. thailandicus* with other *Artocarpus* species. *A. integer* and *A. heterophyllus* were chosen as an outgroup. Two minor clades, the blue and red square dashed line, showed that both species were clustered together in the same clade with low bootstrap support. Some parts of the tree showed unresolved topology, Figure S26: Four combined regions maximum likelihood consensus tree of *A. lacucha* and *A. thailandicus* with other *Artocarpus* species. *A. integer* and *A. heterophyllus* were chosen as an outgroup. Two minor clades, the blue and red square dashed line, showed that both species were clustered together in the same clade with low bootstrap support. Some parts of the tree showed unresolved topology, Figure S27: Four combined regions maximum parsimony (without branches collapsed) tree of *A. lacucha* and *A. thailandicus* with other *Artocarpus* species. *A. integer* and *A. heterophyllus* were chosen as an outgroup, Figure S28: Four combined regions maximum likelihood (without branches collapsed) tree of *A. lacucha* and *A. thailandicus* with other *Artocarpus* species. *A. integer* and *A. heterophyllus* were chosen as an outgroup, Supplementary file S1: Four loci multiple alignment.
