# Supplementary material for: Taxonomic Notes on the ‘Mahat’ (Artocarpus lacucha and A. thailandicus, Moraceae) Species Complex in Thailand
Source: Plants (Basel). 2020 Mar 22;9(3):391. doi: 10.3390/plants9030391 (PMC7154811; doi:10.3390/plants9030391)
Supplement: Supplementary file 1 [file plants-09-00391-s001.zip › Supplementary data.docx]

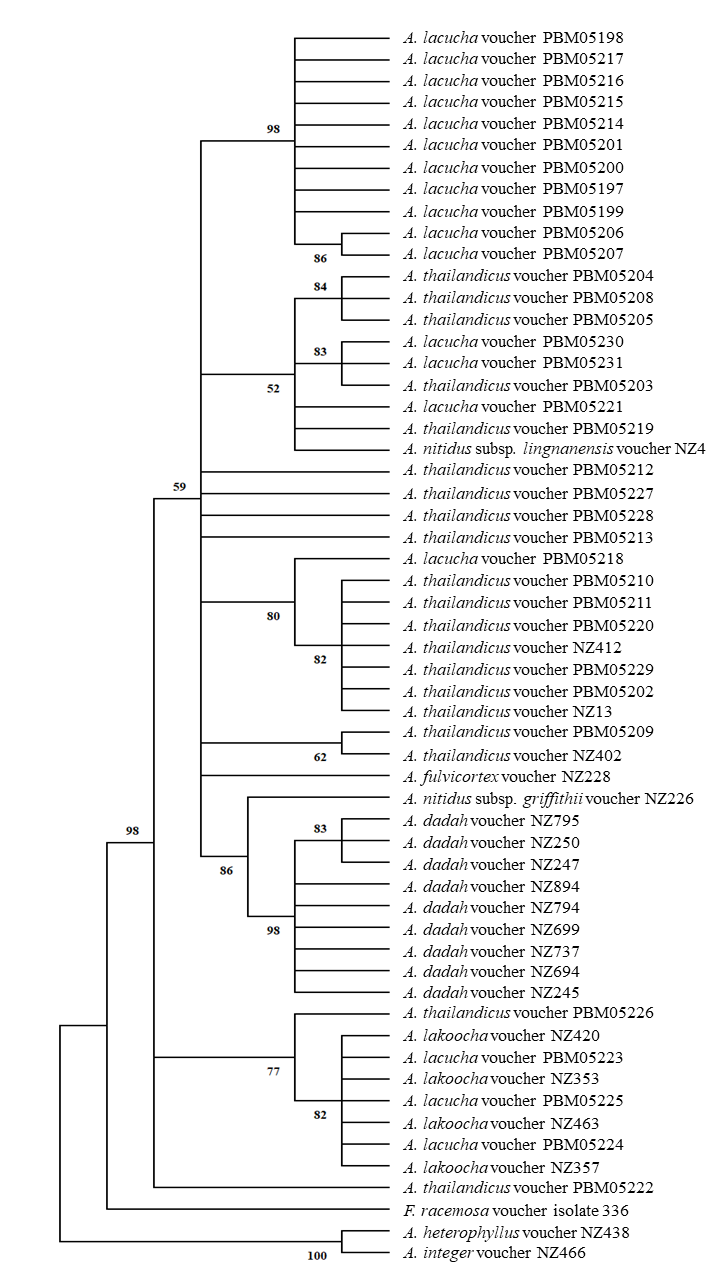


**Figure S1.** ITS region maximum parsimony consensus tree of *A. lacucha* and *A. thailandicus* with other *Artocarpus* species. *A. integer* and *A. heterophyllus* were chosen as an outgroup***.***


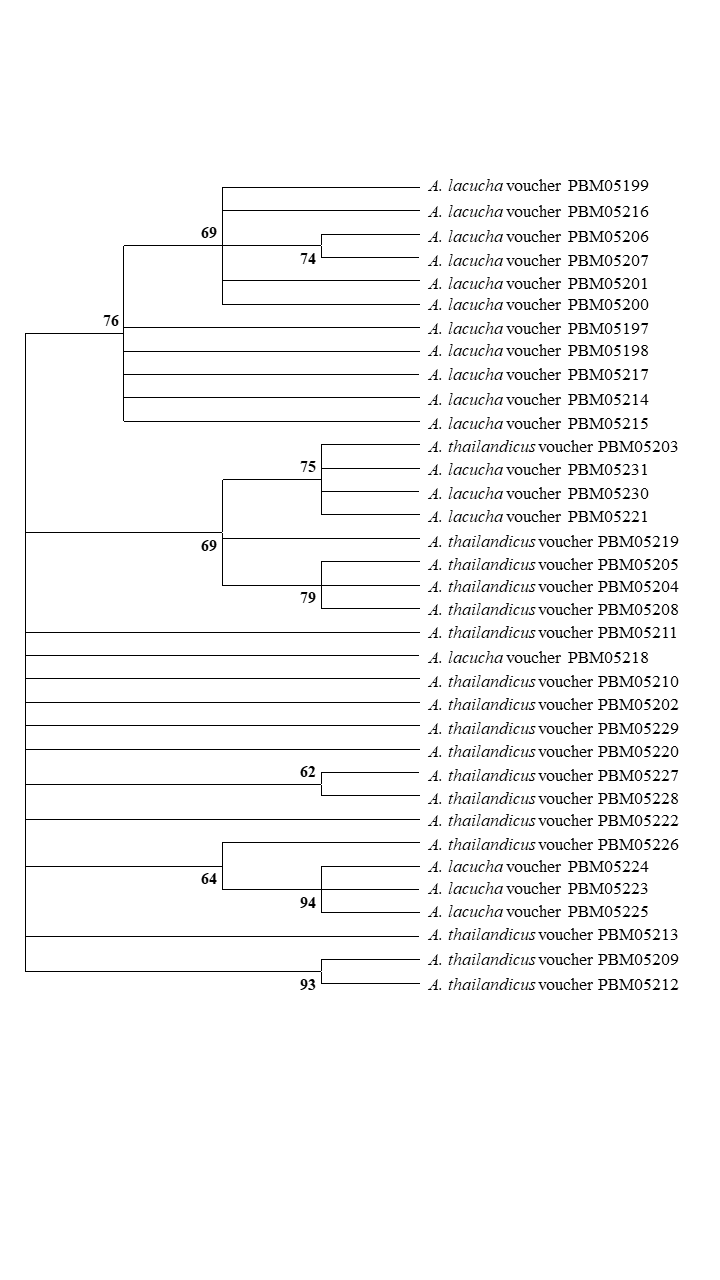


**Figure S2.** ETS region maximum parsimony consensus tree of *A. lacucha* and *A. thailandicus*.


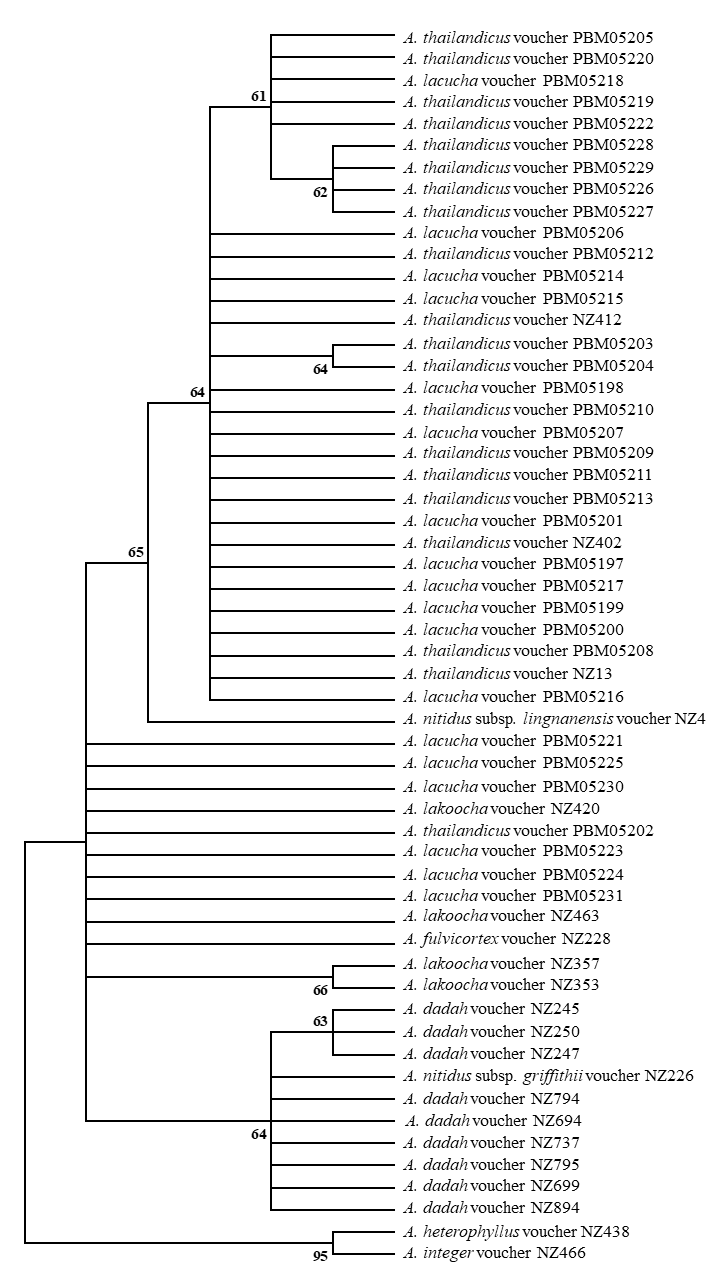


**Figure S3.** *trn*L-F intergenic spacer maximum parsimony consensus tree of *A. lacucha* and *A. thailandicus* with other *Artocarpus* species. *A. integer* and *A. heterophyllus* were chosen as an outgroup*.*


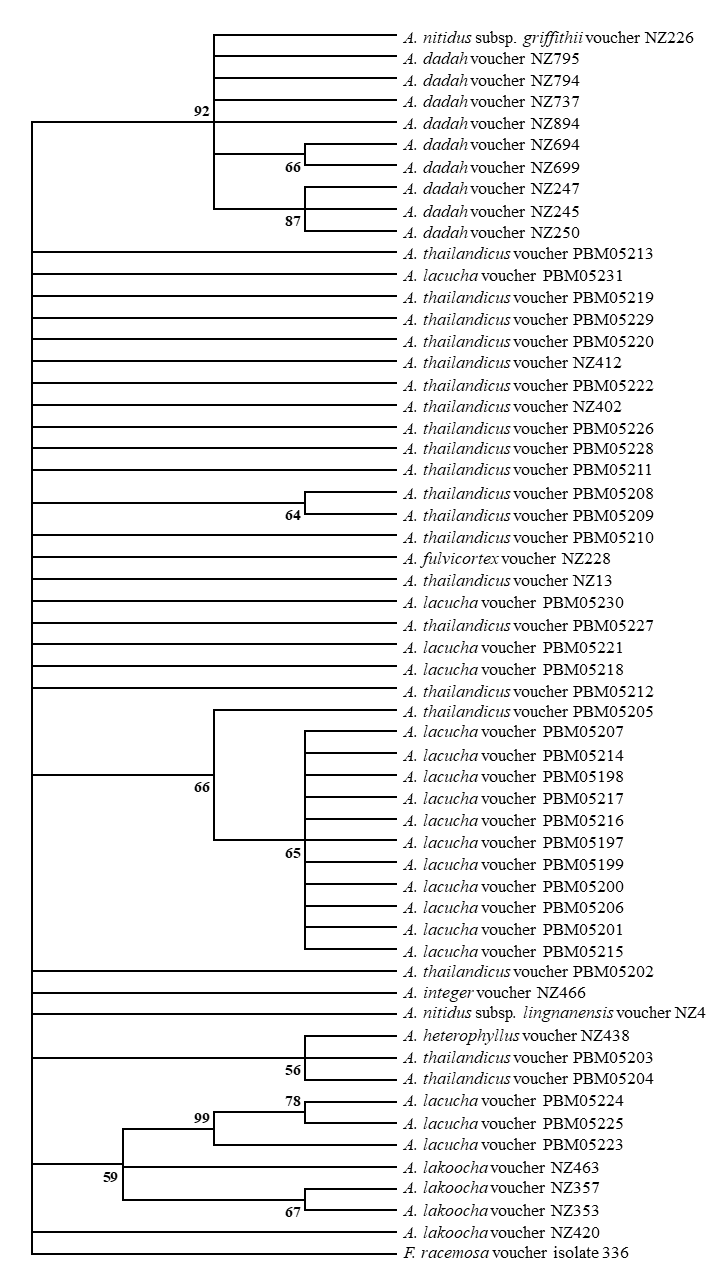


**Figure S4.** *trn*H-*psb*A intergenic spacer maximum parsimony consensus tree of *A. lacucha* and *A. thailandicus* with other *Artocarpus* species. *F. racemosa* was chosen as an outgroup*.*


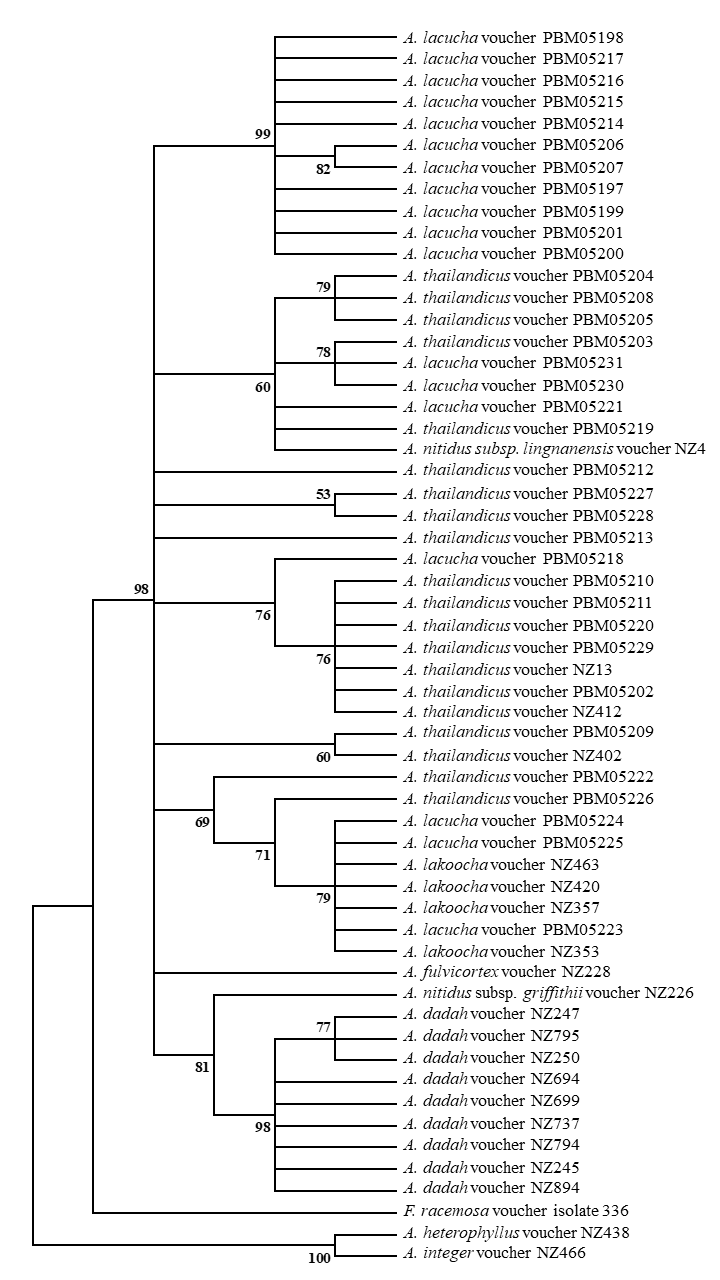


**Figure S5.** Nuclear loci maximum parsimony consensus tree of *A. lacucha* and *A. thailandicus* with other *Artocarpus* species. *A. integer* and *A. heterophyllus* were chosen as an outgroup*.*


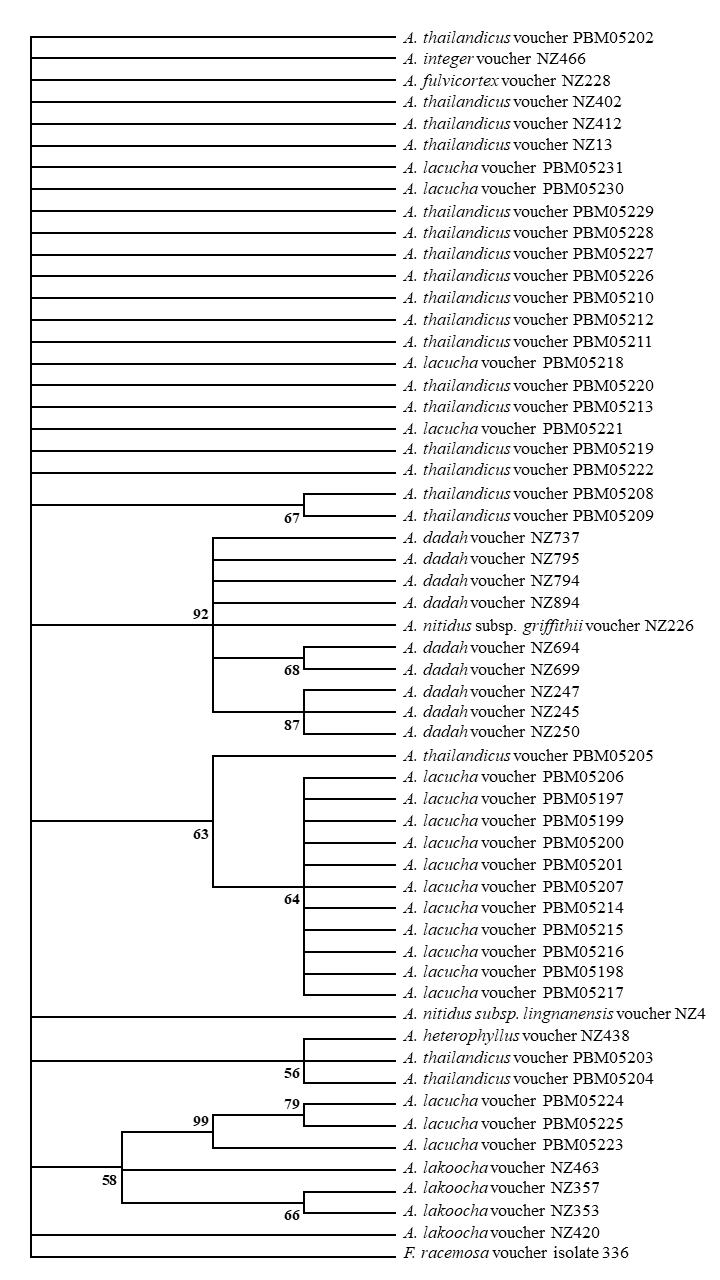


**Figure S6.** Chloroplast loci maximum parsimony consensus tree of *A. lacucha* and *A. thailandicus* with other *Artocarpus* species. *F. racemosa* was chosen as an outgroup*.*


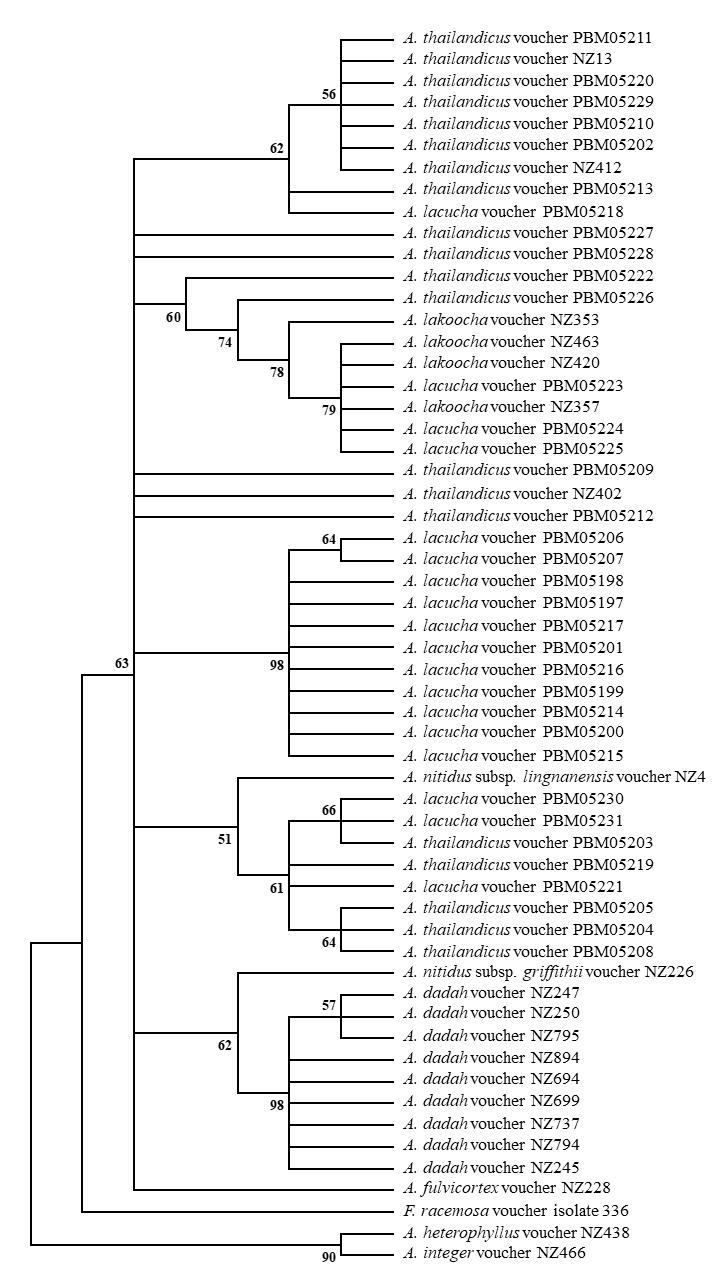


**Figure S7.** ITS region maximum likelihood consensus tree of *A. lacucha* and *A. thailandicus* with other *Artocarpus* species. *A. integer* and *A. heterophyllus* were chosen as an outgroup*.*


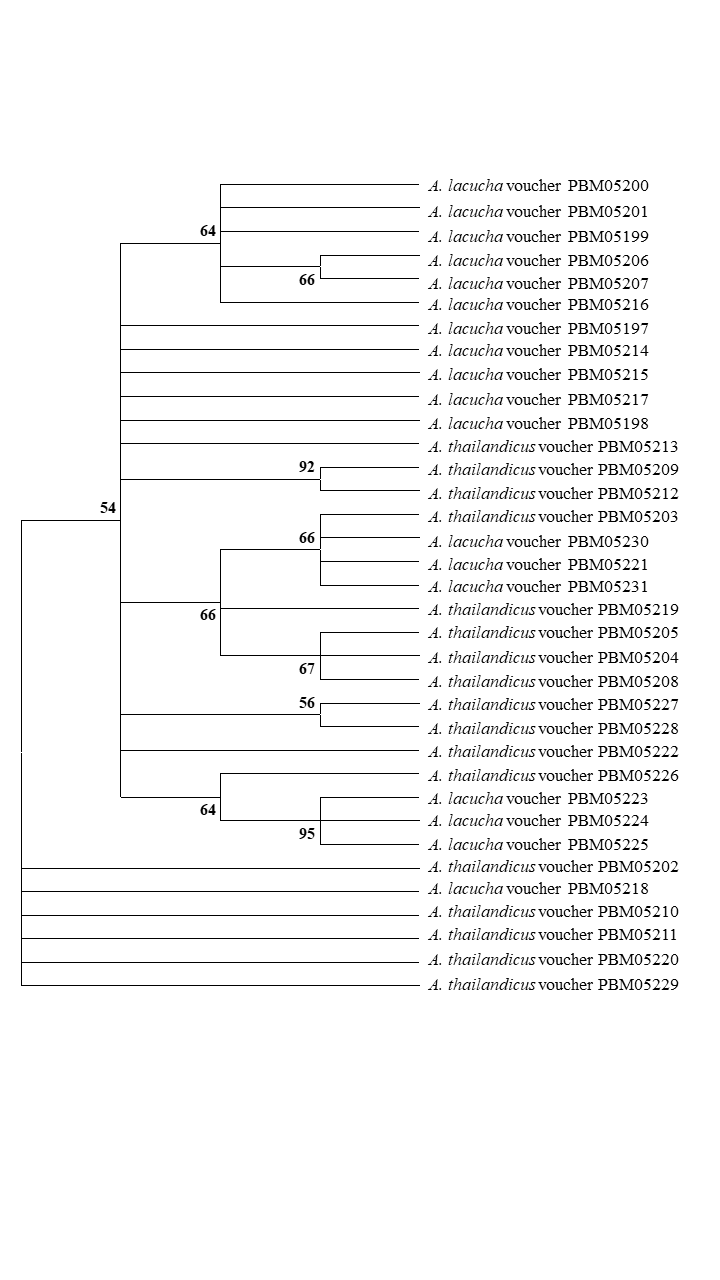


**Figure S8.** ETS region maximum likelihood consensus tree of *A. lacucha* and *A. thailandicus*.


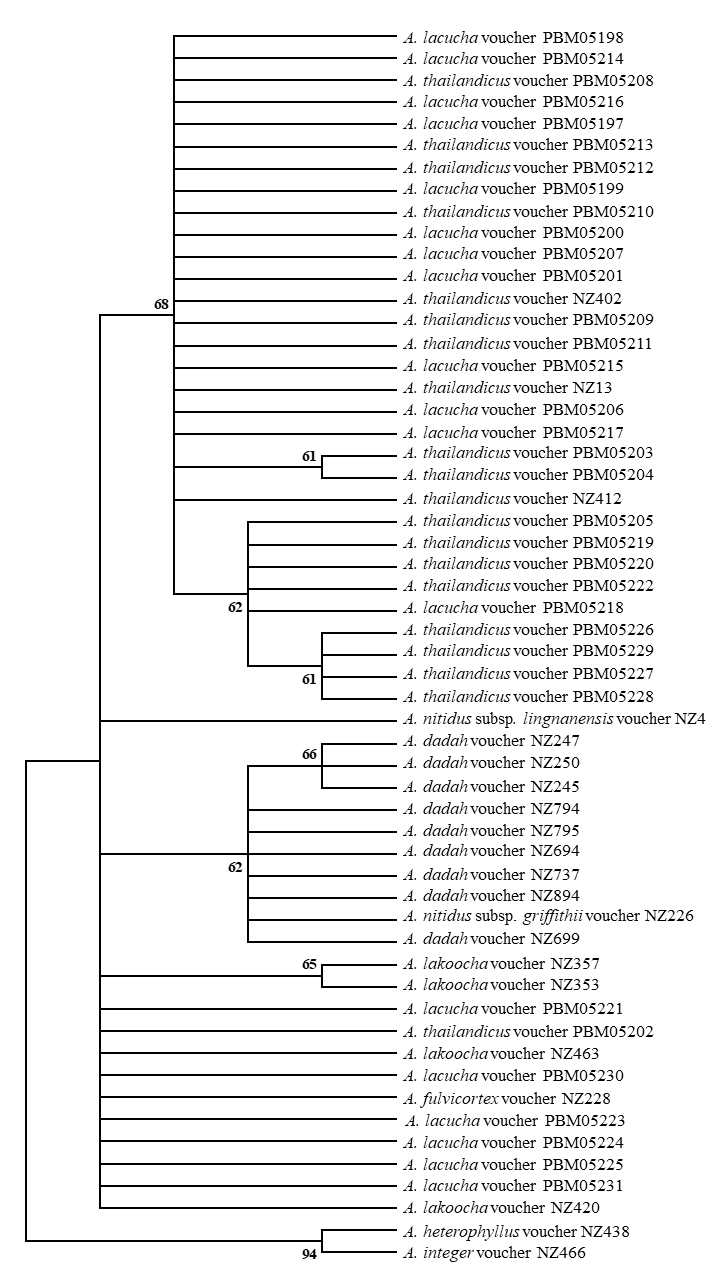


**Figure S9.** *trn*L-F intergenic spacer maximum likelihood consensus tree of *A. lacucha* and *A. thailandicus* with other *Artocarpus* species. *A. integer* and *A. heterophyllus* were chosen as an outgroup***.***


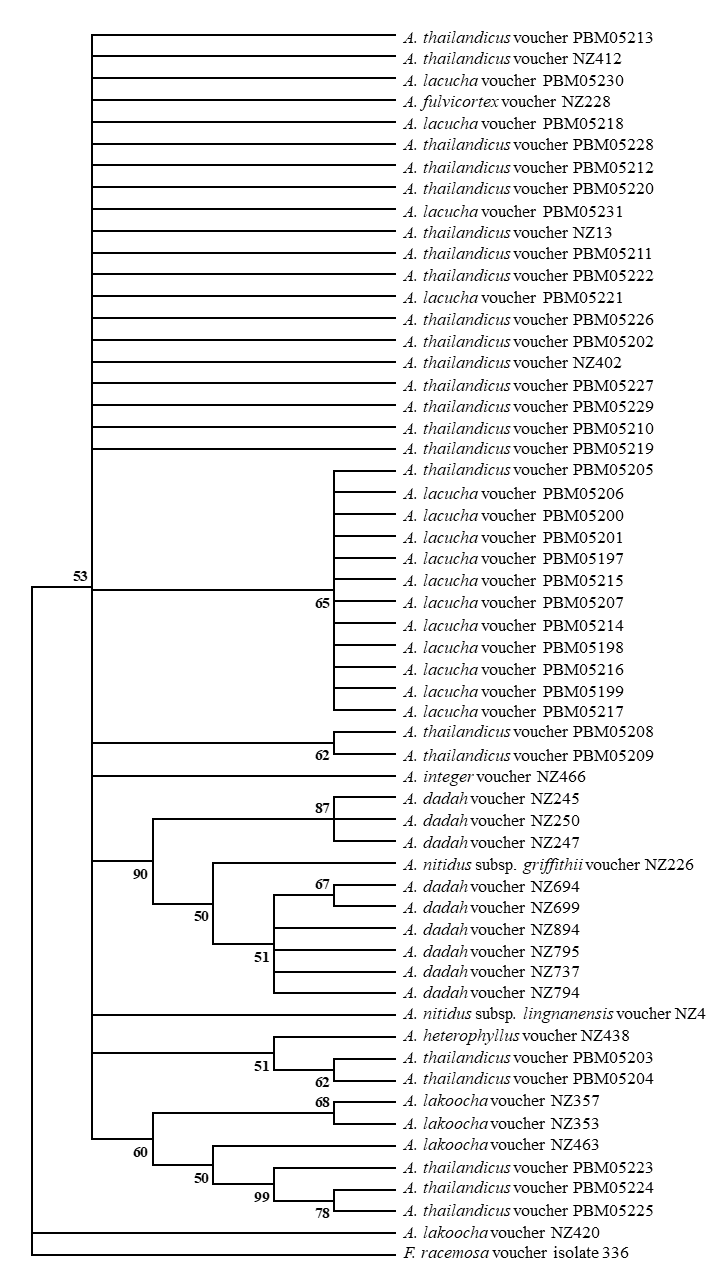


**Figure S10.** *trn*H-*psb*A intergenic spacer maximum likelihood consensus tree of *A. lacucha* and *A. thailandicus* with other *Artocarpus* species. *F. racemosa* was chosen as an outgroup*.*


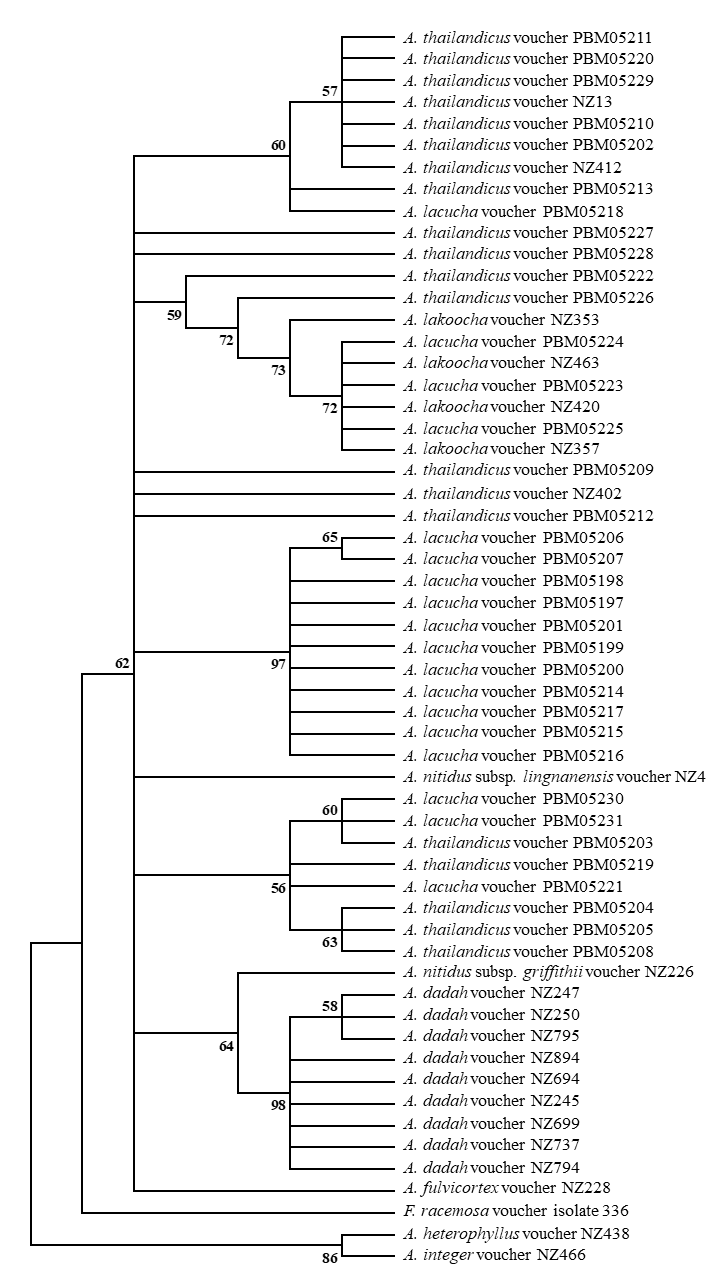


**Figure S11.** Nuclear loci maximum likelihood consensus tree of *A. lacucha* and *A. thailandicus* with other *Artocarpus* species. *A. integer* and *A. heterophyllus* were chosen as an outgroup*.*


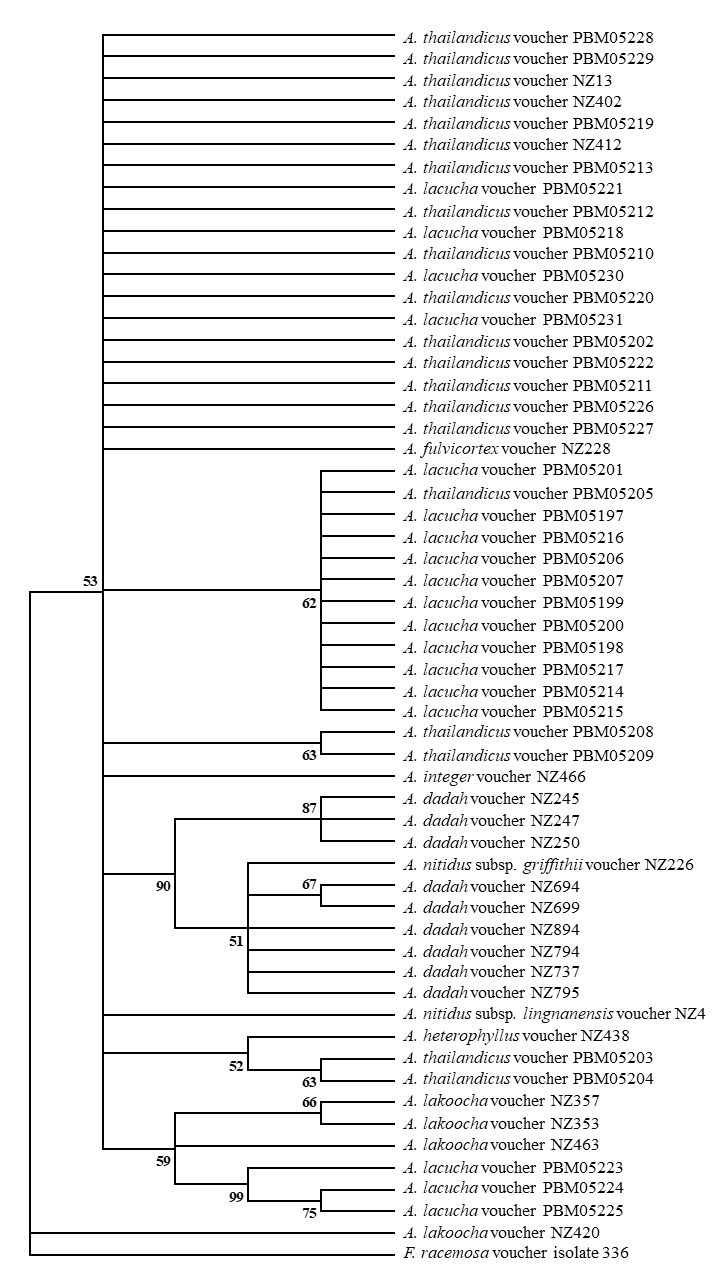


**Figure S12.** Chloroplast loci maximum likelihood consensus tree of *A. lacucha* and *A. thailandicus* with other *Artocarpus* species. *F. racemosa* was chosen as an outgroup*.*


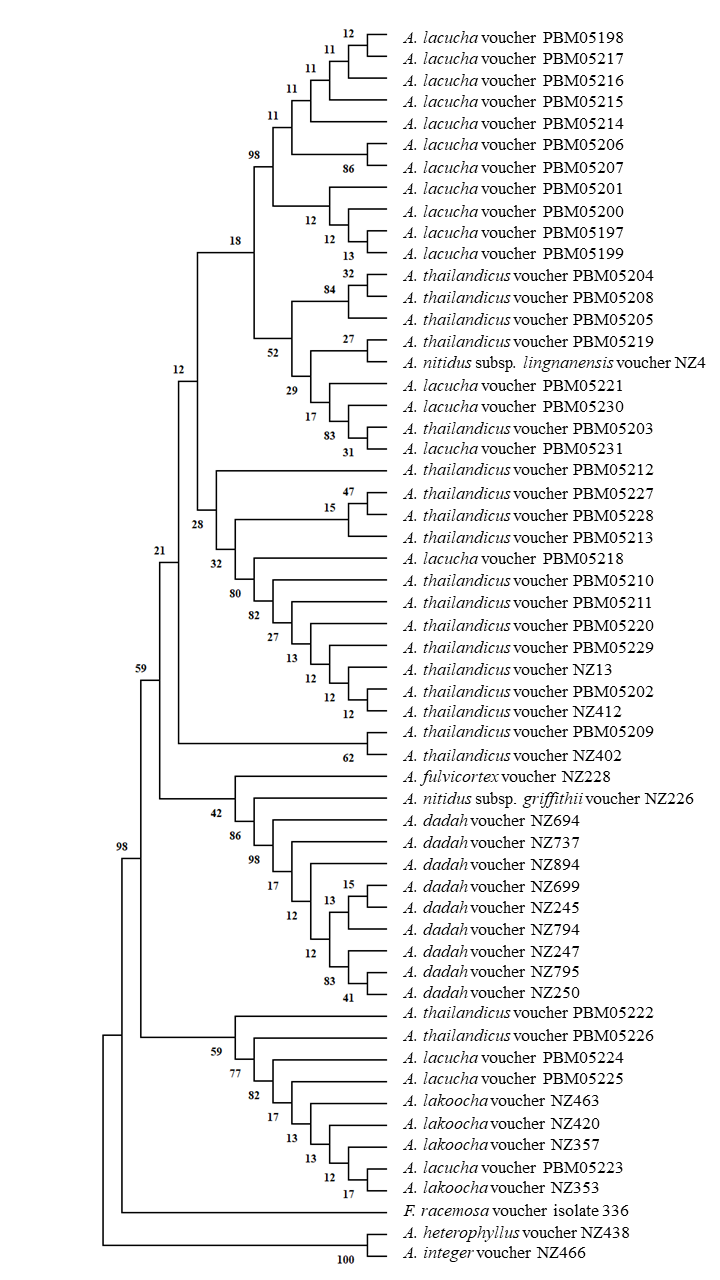


**Figure S13.** ITS region maximum parsimony (without branches collapsed) tree of *A. lacucha* and *A. thailandicus* with other *Artocarpus* species. *A. integer* and *A. heterophyllus* were chosen as an outgroup*.*


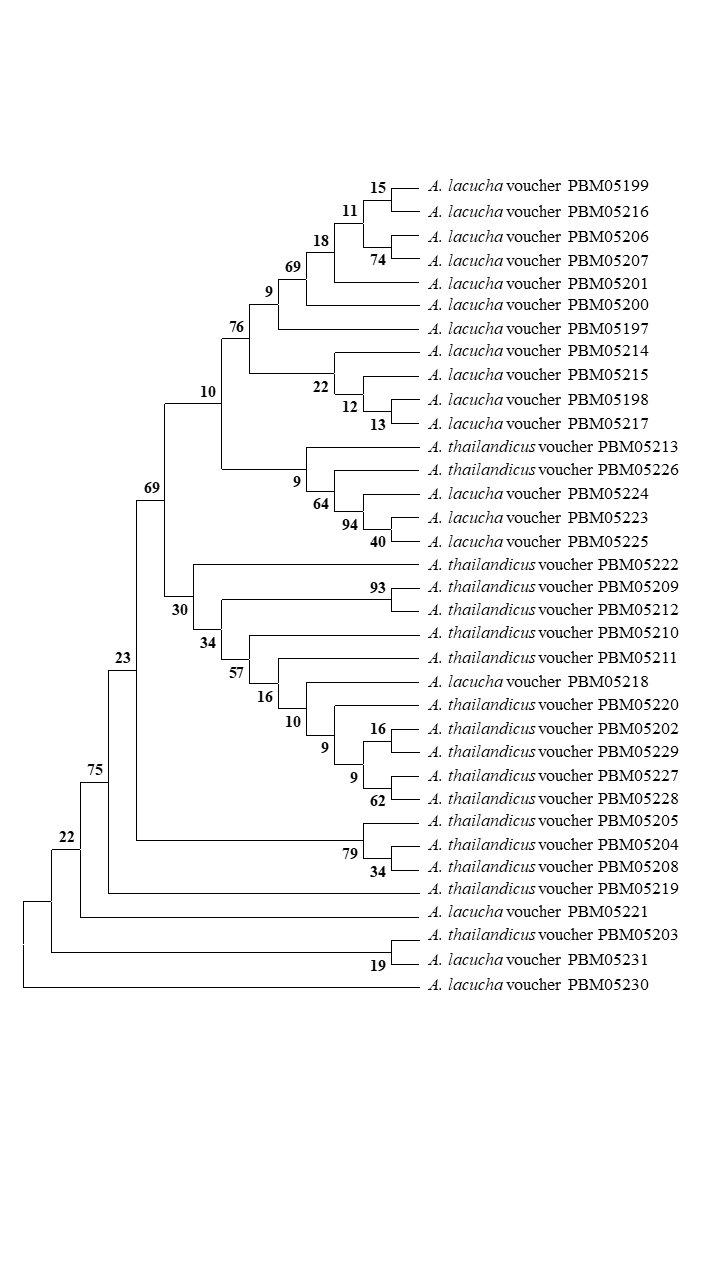


**Figure S14.** ETS region maximum parsimony (without branches collapsed) tree of *A. lacucha* and *A. thailandicus*.


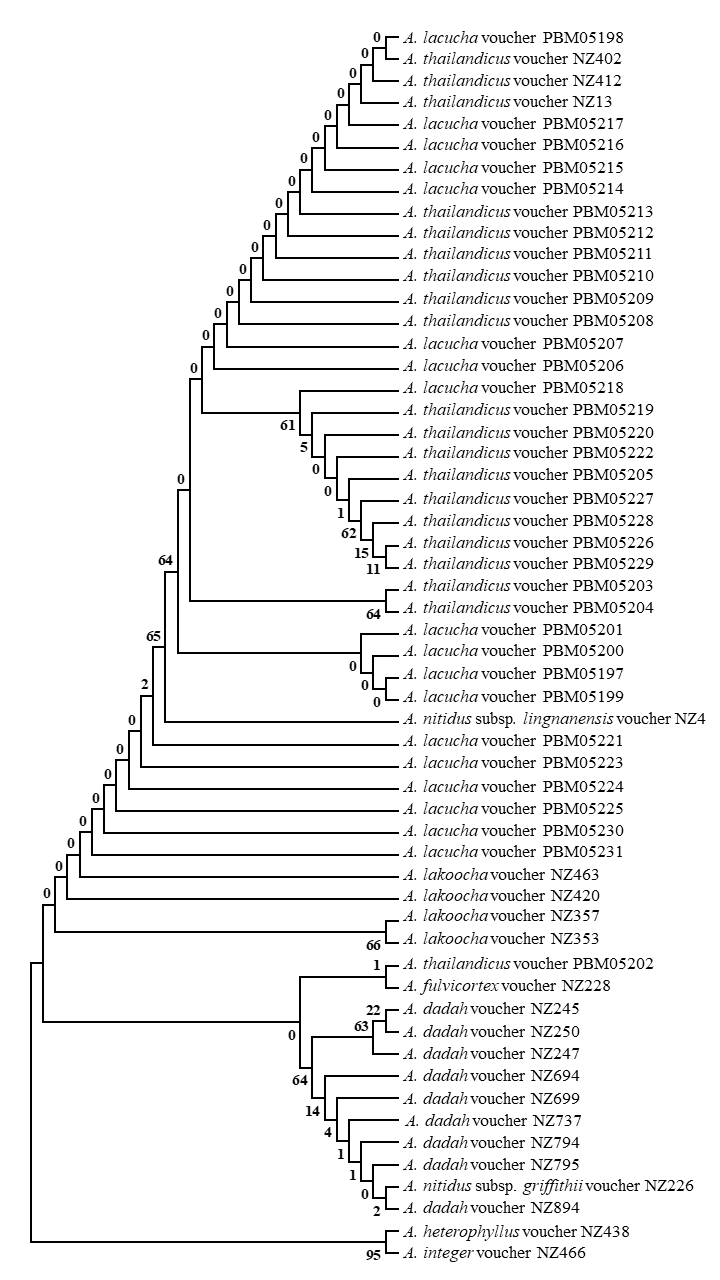


**Figure S15.** *trn*L-F intergenic spacer maximum parsimony (without branches collapsed) tree of *A. lacucha* and *A. thailandicus* with other *Artocarpus* species. *A. integer* and *A. heterophyllus* were chosen as an outgroup*.*


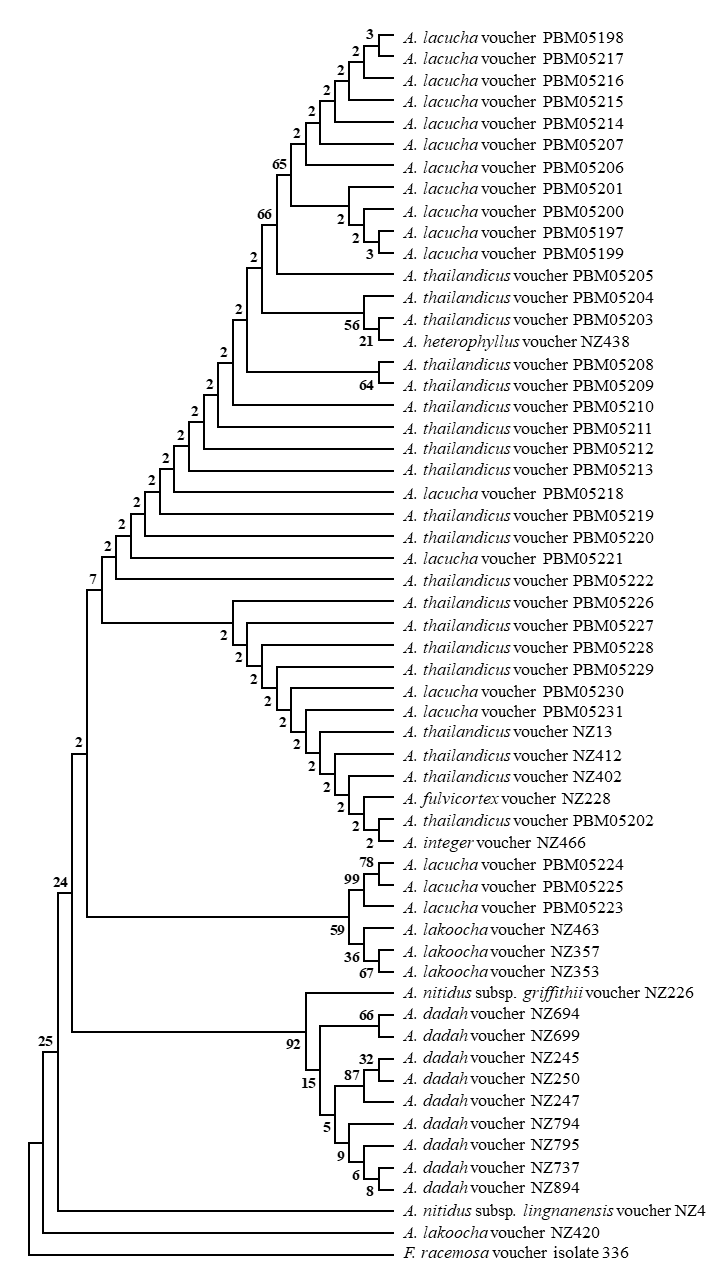


**Figure S16.** *trn*H-*psb*A intergenic spacer maximum parsimony (without branches collapsed) tree of *A. lacucha* and *A. thailandicus* with other *Artocarpus* species. *F. racemosa* was chosen as an outgroup*.*


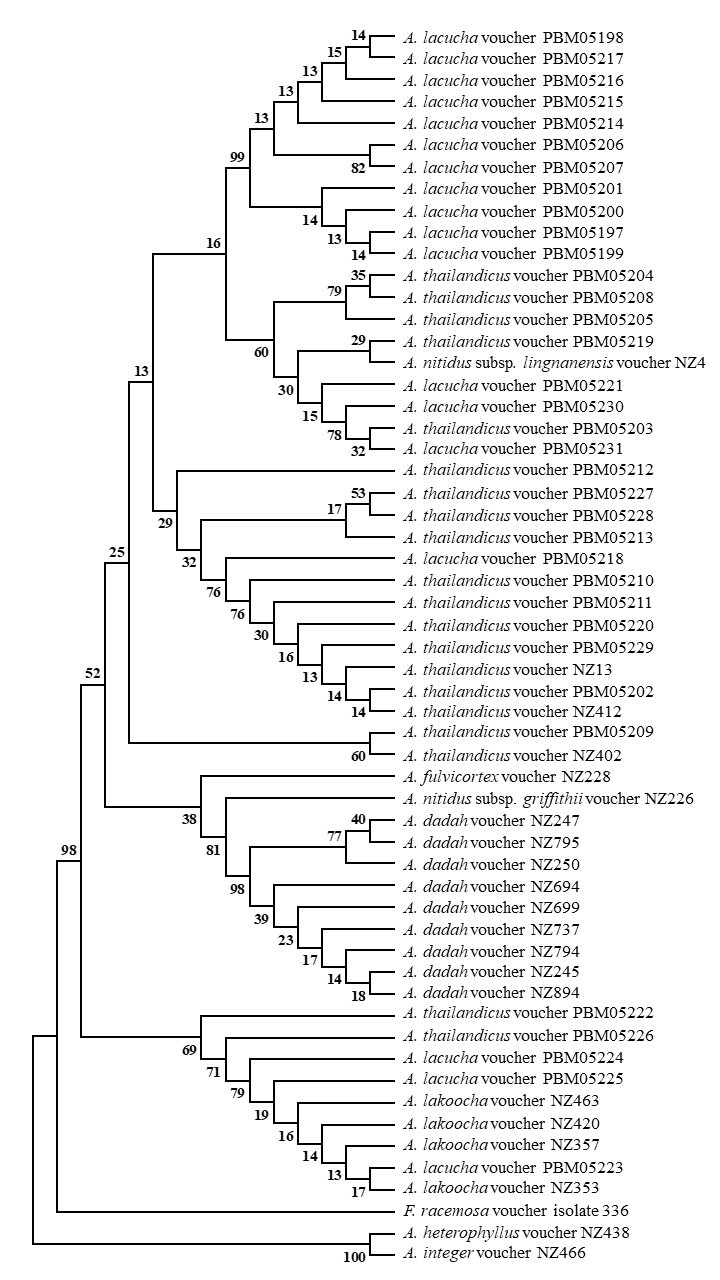


**Figure S17.** Nuclear loci maximum parsimony (without branches collapsed) tree of *A. lacucha* and *A. thailandicus* with other *Artocarpus* species. *A. integer* and *A. heterophyllus* were chosen as an outgroup*.*


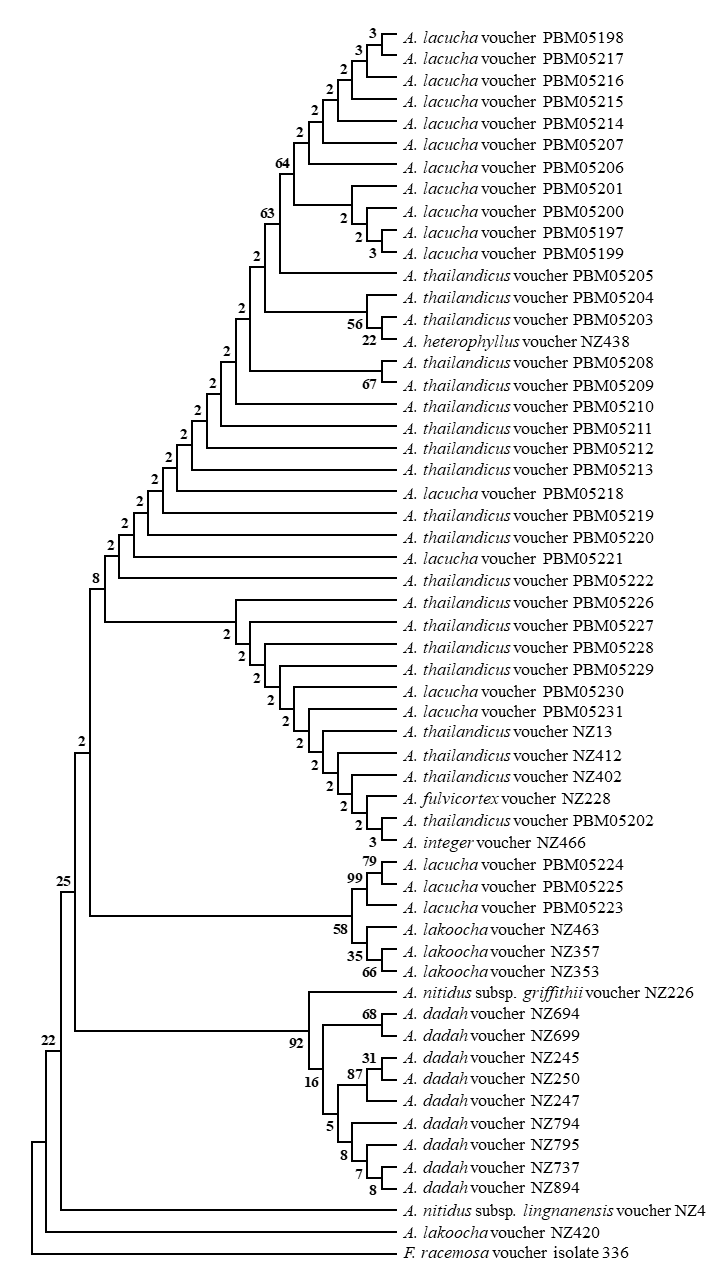


**Figure S18.** Chloroplast loci maximum parsimony (without branches collapsed) tree of *A. lacucha* and *A. thailandicus* with other *Artocarpus* species. *F. racemosa* was chosen as an outgroup*.*


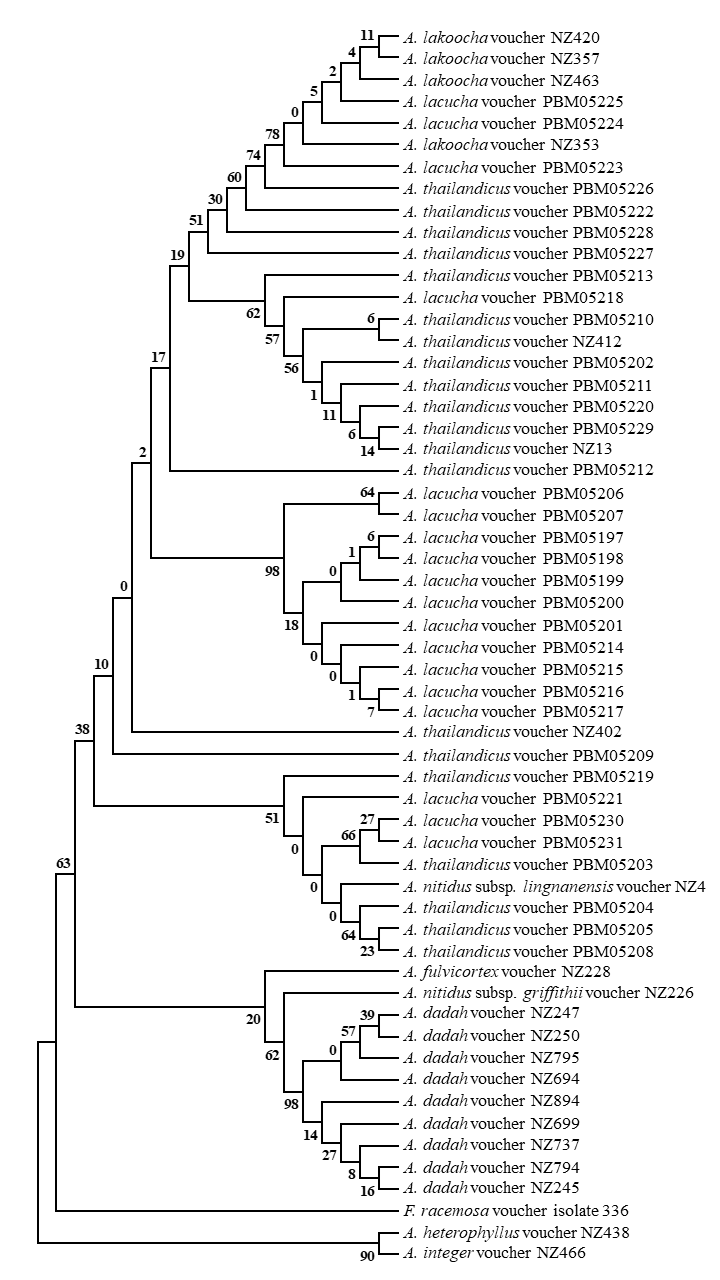


**Figure S19.** ITS region maximum likelihood (without branches collapsed) tree of *A. lacucha* and *A. thailandicus* with other *Artocarpus* species. *A. integer* and *A. heterophyllus* were chosen as an outgroup*.*

##
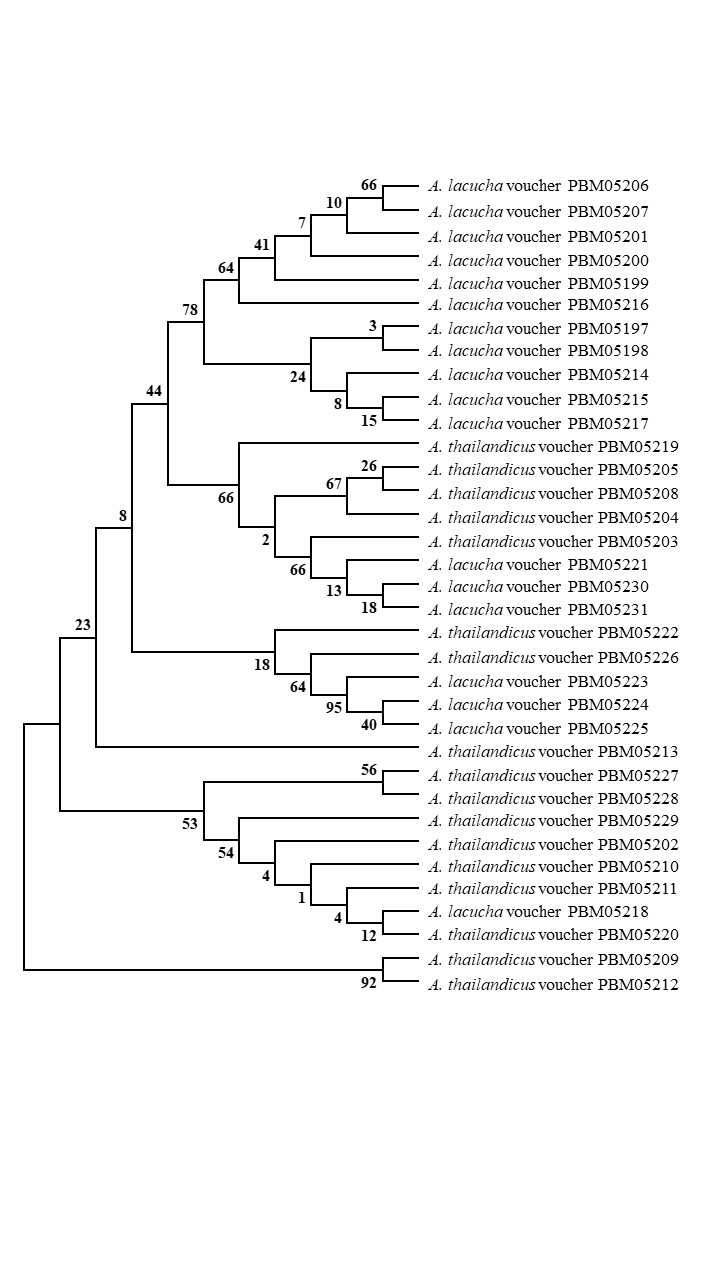


**Figure S20.** ETS region maximum likelihood (without branches collapsed) tree of *A. lacucha* and *A. thailandicus*.


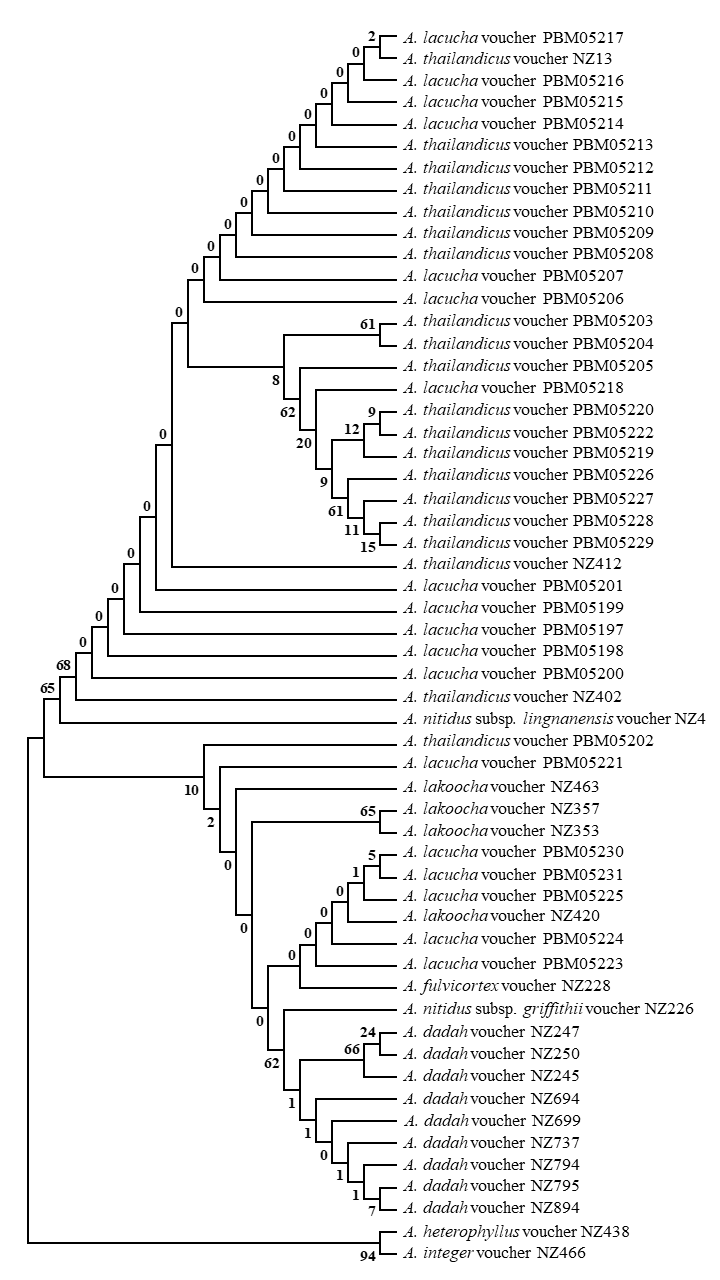


**Figure S21.** *trn*L-F intergenic spacer maximum likelihood (without branches collapsed) tree of *A. lacucha* and *A. thailandicus* with other *Artocarpus* species. *A. integer* and *A. heterophyllus* were chosen as an outgroup*.*


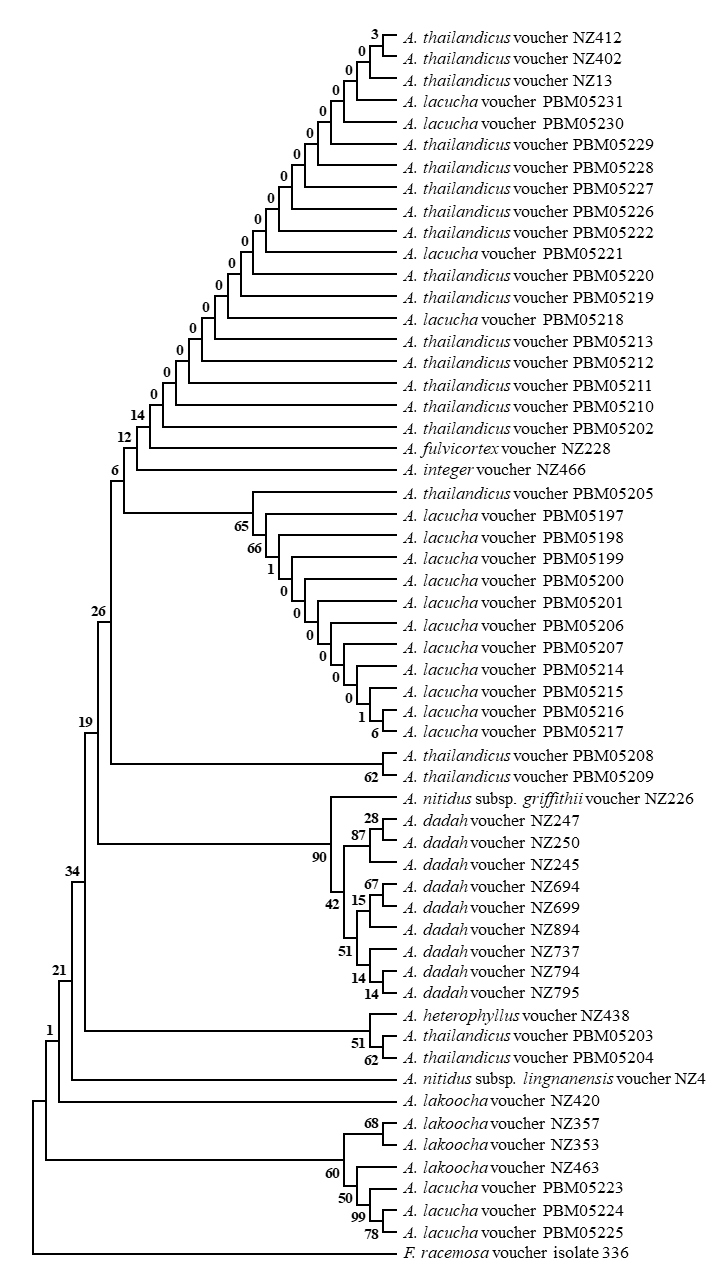


**Figure S22.** *trn*H-*psb*A intergenic spacer maximum likelihood (without branches collapsed) tree of *A. lacucha* and *A. thailandicus* with other *Artocarpus* species. *F. racemosa* was chosen as an outgroup.


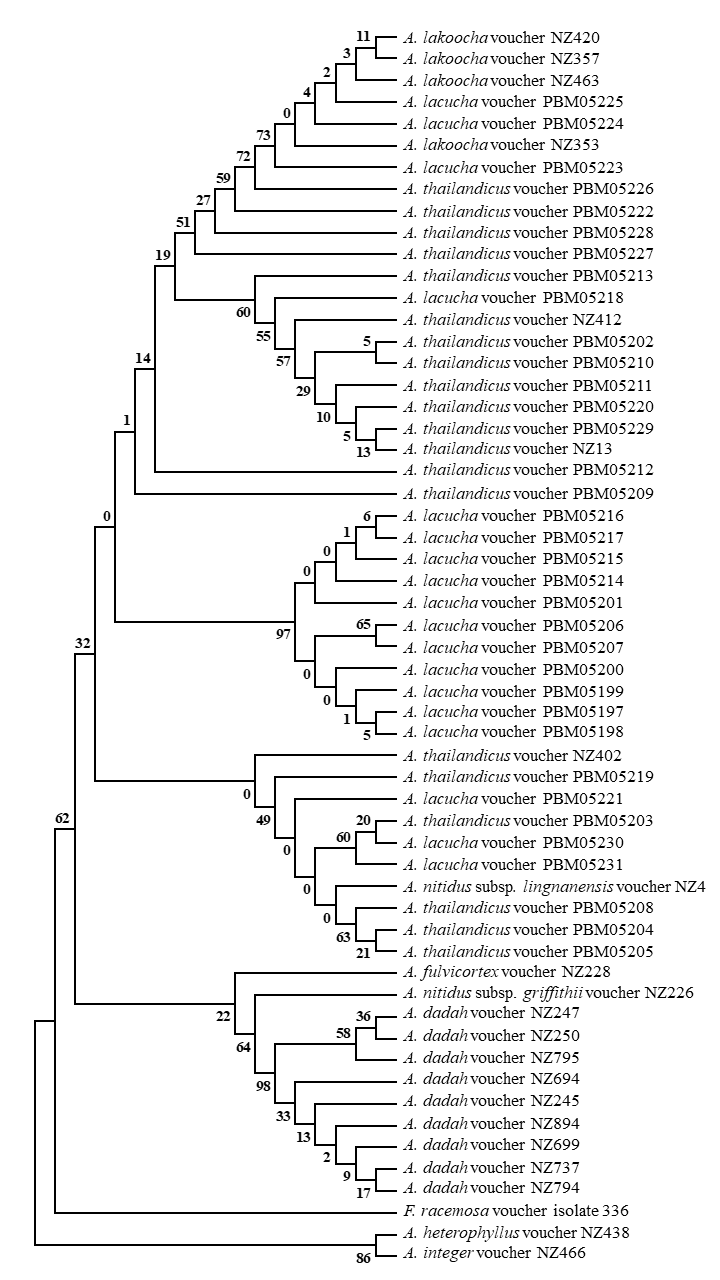


**Figure S23.** Nuclear loci maximum likelihood (without branches collapsed) tree of *A. lacucha* and *A. thailandicus* with other *Artocarpus* species. *A. integer* and *A. heterophyllus* were chosen as an outgroup*.*


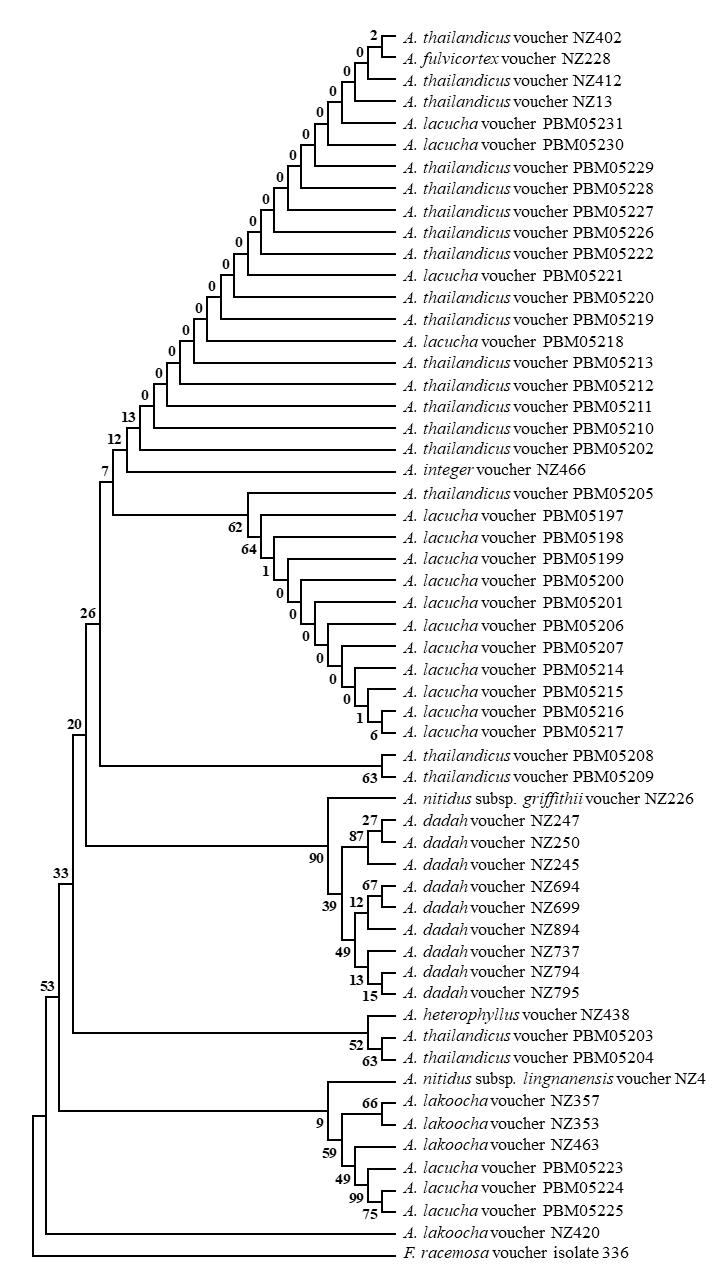


**Figure S24.** Chloroplast loci maximum likelihood (without branches collapsed) tree of *A. lacucha* and *A. thailandicus* with other *Artocarpus* species. *F. racemosa* was chosen as an outgroup*.*


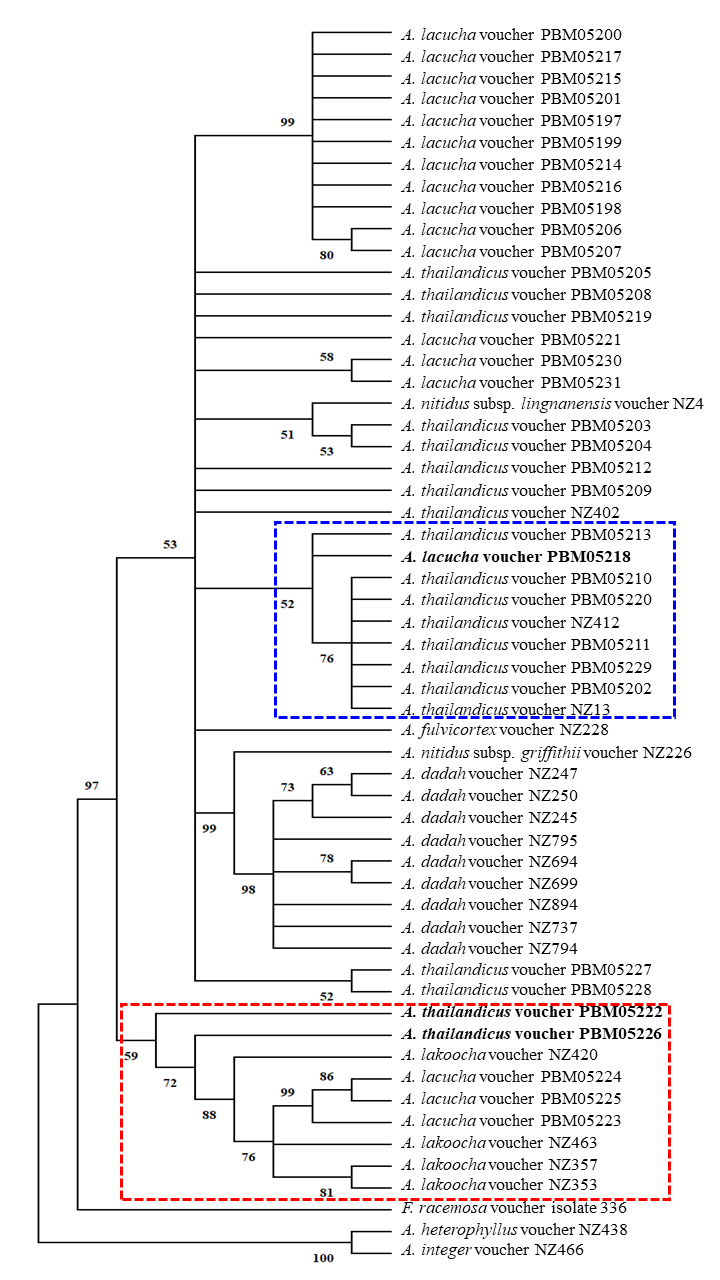


**Figure S25.** Four combined regions maximum parsimony consensus tree of *A. lacucha* and *A. thailandicus* with other *Artocarpus* species. *A. integer* and *A. heterophyllus* were chosen as an outgroup*.* Two minor clades, the blue and red square dashed line, showed that both species were clustered together in the same clade with low bootstrap support. Some parts of the tree showed unresolved topology.

**
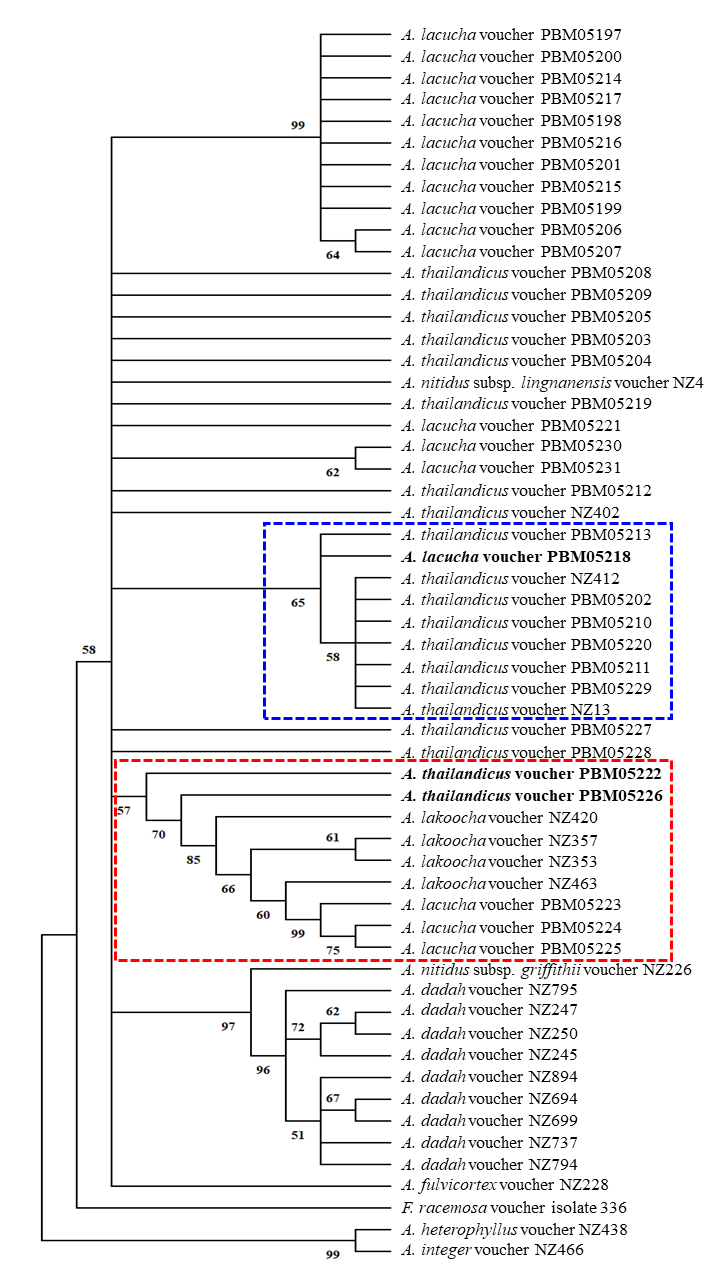
**

**Figure S26.** Four combined regions maximum likelihood consensus tree of *A. lacucha* and *A. thailandicus* with other *Artocarpus* species. *A. integer* and *A. heterophyllus* were chosen as an outgroup*.* Two minor clades, the blue and red square dashed line, showed that both species were clustered together in the same clade with low bootstrap support. Some parts of the tree showed unresolved topology.


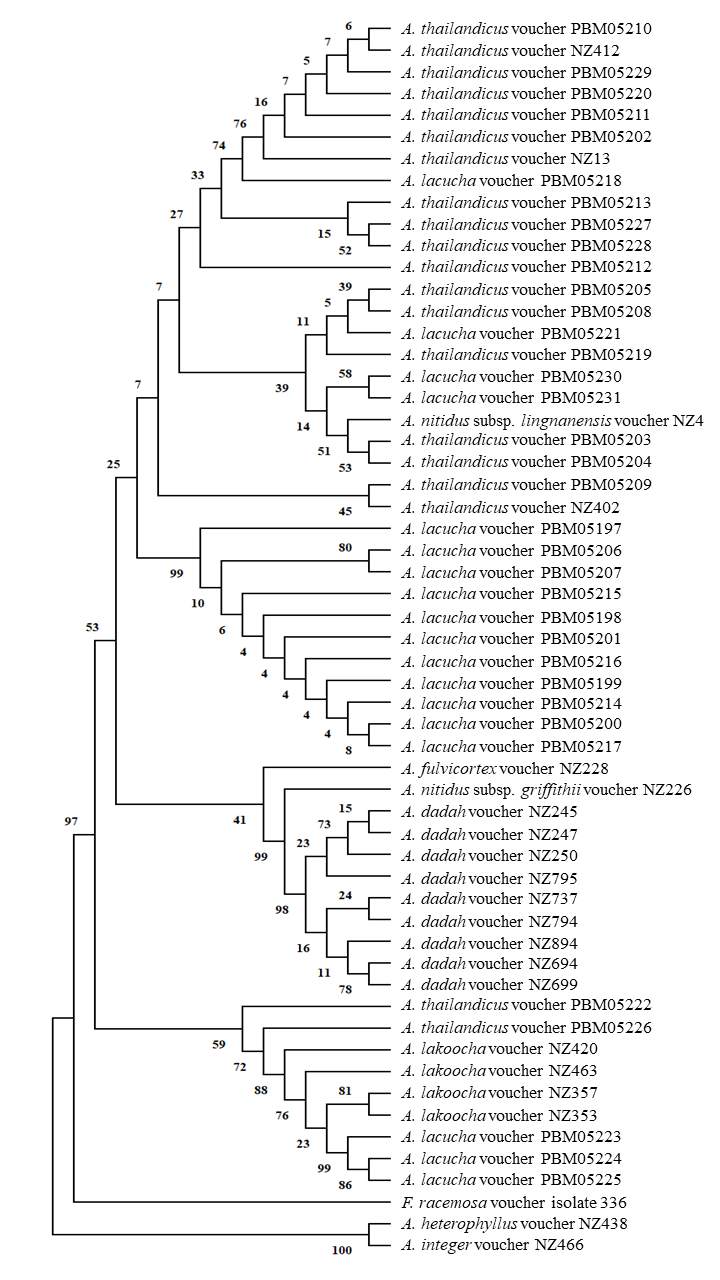


**Figure S27.** Four combined regions maximum parsimony (without branches collapsed) tree of *A. lacucha* and *A. thailandicus* with other *Artocarpus* species. *A. integer* and *A. heterophyllus* were chosen as an outgroup*.*


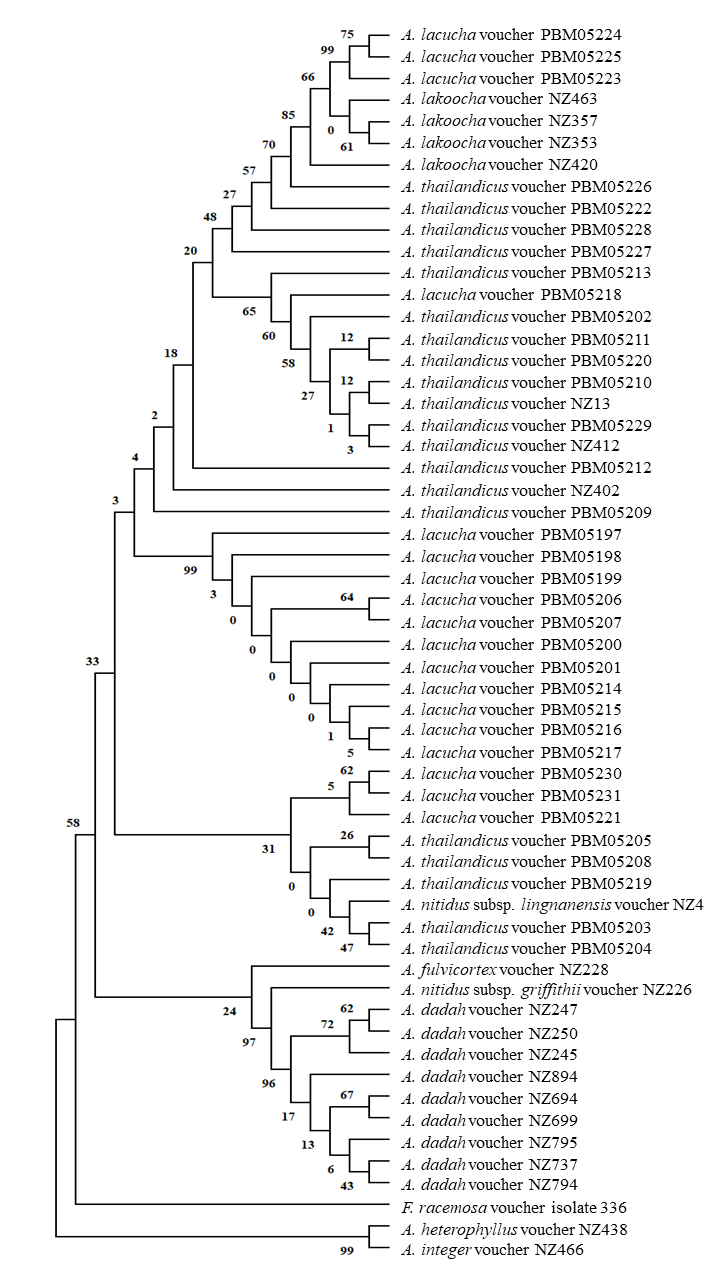


**Figure S28.** Four combined regions maximum likelihood (without branches collapsed) tree of *A. lacucha* and *A. thailandicus* with other *Artocarpus* species. *A. integer* and *A. heterophyllus* were chosen as an outgroup*.*
